# Age-stratified hormonal profiles in adolescent and young adult PCOS/PMOS: higher mid-adolescent AMH largely unexplained by BMI

**DOI:** 10.3389/fendo.2026.1890066

**Published:** 2026-08-05

**Authors:** Sait Erbey, Murat Polat, Bilge Erbey, İnci Kahyaoğlu

**Affiliations:** 1 Department of Obstetrics and Gynaecology, Ankara Etlik City Hospital, Ankara, Türkiye; 2 Independent Obstetrics and Gynaecology Specialist, Ankara, Türkiye

**Keywords:** adolescent, anti-Müllerian hormone, body mass index, hirsutism, hyperandrogenism, LH/FSH ratio, PMOS, polycystic ovary syndrome

## Abstract

Polycystic ovary syndrome (PCOS) — for which a global multistakeholder consensus has recently proposed the name polyendocrine metabolic ovarian syndrome (PMOS) — shows phenotypic variation across adolescence that is poorly characterised in Turkish populations, and because puberty raises both body mass index (BMI) and reproductive hormones in parallel, age and BMI effects are potentially confounded. Within a single-centre retrospective cohort of clinician-assigned PCOS/PMOS (January 2024–February 2026), we described how hormonal, anthropometric and clinical profiles differ across age and whether these differences are explained by the parallel BMI gradient; because the cohort has no healthy controls, all analyses describe intra-PCOS variation and cannot establish PCOS-specificity. Of 1,228 patients with a clinician-assigned PCOS diagnosis among 11,324 adolescent visits, 878 met inclusion criteria (94.5% fulfilled ≥2 Rotterdam criteria *post hoc*; 40.8% of those ≤19 years met the stricter 2023 ESHRE adolescent criteria). Early-follicular-phase samples (cycle days 2–5; progesterone withdrawal in anovulatory patients) were analysed across four age groups (≤15, n=100; 16–17, n=217; 18–19, n=416; ≥20, n=145), with eight pre-specified sensitivity analyses. Overweight/obesity rose across age groups (35.0%→66.9%) but BMI correlated only weakly with the primary hormonal markers. Oligomenorrhoea/amenorrhoea predominated (78.0%); hirsutism (mFG ≥8) affected 13.0%. AMH (ϵ²=0.203, 95% CI 0.160–0.256), LH/FSH ratio (ϵ²=0.098), total testosterone (ϵ²=0.064) and DHEA-S (ϵ²=0.094) all reached their highest median values in mid-adolescents (16–17 years; all p<0.001). The higher mid-adolescent AMH values were the most robust signal, persisting after BMI-adjusted multivariable median regression and within both the strict Phenotype A and the non-hyperandrogenic Phenotype D (ϵ²=0.197) subsets. Total testosterone was the only significant correlate of hirsutism (OR 1.070 per ng/dL, 95% CI 1.055–1.086; AUROC 0.818, optimism-corrected 0.815) after age and BMI adjustment; because SHBG and free androgen index were unavailable, this reflects the strongest correlate within the available biochemical panel rather than a complete androgen assessment. Oestradiol showed the largest age-related effect overall (ϵ²=0.390), consistent with progressive pubertal oestrogenisation. In this cross-sectional, control-free cohort, BMI did not materially explain the age-related hormonal variation; findings are hypothesis-generating regarding pubertal (HPO-axis) mechanisms and do not demonstrate within-individual maturation or PCOS-specificity. The P5–P95 distributions represent intra-PCOS benchmarks, not population norms.

## Introduction

Polycystic ovary syndrome (PCOS) affects an estimated 6–21% of women of reproductive age and is the most common endocrine disorder in this population (1–4). Following the completion of data collection and the ethics approval for the present study, a global multistakeholder consensus process led by Teede and colleagues recommended renaming PCOS to polyendocrine metabolic ovarian syndrome (PMOS), with a planned 3-year managed transition period and integration into the 2028 International Guideline update (5). As the present study was conceived, designed, and ethically approved under the established PCOS nomenclature (approval reference AEŞH-BADEK1-2026-425, 6 May 2026), as all included patients carried a clinician-assigned PCOS diagnosis throughout the study period (January 2024 – February 2026), and as the prevailing 2023 ESHRE international evidence-based guideline used to define our reference subset retains the PCOS terminology, we retain the term PCOS throughout this manuscript while acknowledging the forthcoming transition. The syndrome carries substantially elevated lifetime risk of type 2 diabetes mellitus, metabolic syndrome, non-alcoholic fatty liver disease, and cardiovascular disease (6, 7). Among the many factors that modulate the PCOS phenotype, body mass index (BMI) occupies a particularly central position: obesity independently amplifies hyperandrogenism, insulin resistance, and LH pulsatility, and has been identified as a major driver of interpatient hormonal variability (8, 9). Yet BMI itself rises substantially during normal pubertal development, meaning that in adolescent PCOS cohorts, the effects of age and BMI on the hormonal phenotype are often co-directional and potentially confounded.

The presenting clinical complaints of adolescent PCOS — oligomenorrhoea, amenorrhoea, dysmenorrhoea, hirsutism, and pelvic pain — overlap extensively with the normal physiology of puberty during the first two to three years after menarche (10–12). The hypothalamic–pituitary–ovarian (HPO) axis undergoes profound maturation between ages 12 and 20 years, with LH pulse amplitude and frequency peaking in the peri-menarche period and normalising as hypothalamic negative feedback matures (13). Adult-derived laboratory thresholds may therefore be misleading when applied to adolescent patients (14, 15).

The 2023 International Evidence-based Guideline for PCOS, jointly developed by ESHRE and Monash University, recommends deferring a definitive adolescent PCOS diagnosis until at least two years post-menarche and cautions against applying adult AMH thresholds to adolescents (15). AMH declines physiologically with age within the PCOS phenotype, and BMI exerts an additional independent negative effect (16). Disentangling these influences requires age-stratified data with concomitant BMI information — data that are largely absent from the Turkish literature. A targeted PubMed search (queried 9 May 2026; combinations of ‘Turkish/Turkey’, ‘PCOS/polycystic ovary syndrome’, ‘adolescent’, ‘young adult’, and ‘AMH/hormonal profile/age-stratified’) identified no published Turkish adolescent PCOS cohort exceeding 100 PCOS cases, and no cohort providing age-stratified hormonal centile data within PCOS patients across the adolescent and young adult range.

The present study was designed to provide a comprehensive characterisation of hormonal, anthropometric, and clinical profiles in the largest single-centre age-stratified adolescent and young adult PCOS cohort from Türkiye (n=878), with explicit attention to the role of BMI as a potential confounder. Because the cohort lacks healthy controls, all findings describe intra-PCOS variation by age. The largest age-related effect in the cohort was for estradiol (ϵ²=0.390), consistent with progressive normal pubertal oestrogenisation; we therefore cannot determine whether the mid-adolescent hormonal pattern is specific to PCOS or reflects normal puberty amplified in PCOS. Group 4 (≥20 yr, n=145) includes young adults and was retained as a reference group for the post-adolescent hormonal profile.

## Materials and methods

This was a single-centre retrospective cohort study at the Adolescent Outpatient Clinic of Ankara Etlik City Hospital, Ankara, Türkiye. The study was approved by the Clinical Research Ethics Committee of Ankara Etlik City Hospital (approval reference: AEŞH-BADEK1-2026-425; approval date: 6 May 2026) in conformity with the Declaration of Helsinki. This was a retrospective study of existing clinical records; ethics approval was obtained before any data were extracted from the Hospital Information System, and the study period January 2024–February 2026 denotes the dates of the clinical encounters whose records were reviewed, not a prospective enrolment period. The requirement for individual informed consent was waived by the ethics committee given the retrospective, anonymised nature of the data. Patient identifiers were replaced with anonymous numeric codes prior to analysis. Reporting conforms to the Strengthening the Reporting of Observational Studies in Epidemiology (STROBE) checklist for cohort studies (Supplementary File 1: STROBE Checklist) (17). Because the study uses routinely collected health data, reporting additionally conforms to the RECORD extension of STROBE (Supplementary File 2: RECORD Checklist).

The source population comprised 11,324 consecutive patients attending the Adolescent Outpatient Clinic during the study period. Of these, 1,228 (10.8%) received a clinician-assigned PCOS diagnosis (ICD-10 E28.2) per modified Rotterdam and 2023 ESHRE criteria. Inclusion criteria were: age 10–26 years; PCOS confirmed at the index visit; complete hormonal laboratory panel in the Hospital Information System (HIS) within ±90 days of diagnosis; and BMI calculable from weight and height recorded at the same visit. The ±90-day window was applied only for record linkage; all samples were drawn in the standardised early follicular phase, so cycle-phase variability therefore does not account for observed inter-individual differences in hormone concentrations. From the 1,228 PCOS-diagnosed patients, 350 were excluded: incomplete laboratory panel (AMH or ≥3 hormonal parameters missing, n=156), BMI not calculable owing to missing weight or height (n=87), actively treated thyroid disorder (n=34), missing age or date of diagnosis (n=28), treated hyperprolactinaemia (n=18), baseline 17-OHP >2.5 ng/mL, or 2.0–2.5 ng/mL accompanied by additional clinical features warranting endocrine work-up for non-classic congenital adrenal hyperplasia (n=12), primary ovarian insufficiency (n=7), systemic corticosteroids within three months (n=5), age outside the 10–26 yr range (n=2), and one duplicate record removed. This yielded the final analytic cohort of 878 unique patients (**Supplementary Figure 1**).

A *post-hoc* criterion-level verification of the PCOS diagnosis was performed for the entire analytic cohort using the three Rotterdam criteria as operationalised for this dataset: (i) oligo-/amenorrhoea (as documented in HIS); (ii) clinical or biochemical hyperandrogenism (modified Ferriman–Gallwey score ≥8 or total testosterone >55 ng/dL); and (iii) polycystic ovarian morphology (PCOM) on transabdominal or transvaginal ultrasound as reported by the attending clinician. Patients were classified as meeting the Rotterdam criteria when ≥2 of these 3 criteria were satisfied, and assigned to Rotterdam phenotypes A–D accordingly. This approach reflects real-world clinical practice at a tertiary adolescent outpatient clinic, with *post-hoc* criterion-level verification providing an additional layer of diagnostic robustness.

Patients were allocated to four age subgroups (15, 18): Group 1, early adolescent (≤15 yr, n=100); Group 2, mid-adolescent (16–17 yr, n=217); Group 3, late adolescent (18–19 yr, n=416); Group 4, young adult (≥20 yr, n=145). Diagnostic classification in Group 1 was accepted per treating physician documentation; eight patients aged 12–13 years may not have fulfilled the 2-year post-menarche criterion. Age at menarche was recorded for all 878 patients as part of the routine adolescent gynaecology intake history, and gynaecologic age (chronological age minus age at menarche) was derived for each patient; descriptive distributions are reported in **Table 1**, and a pre-specified gynaecologic-age sensitivity analysis is described below. Tanner stage was not systematically retrievable from the Hospital Information System.

**Table 1:** Demographic, anthropometric, and clinical characteristics by age subgroup.

Variable	Group 1 (≤15 yr) n=100	Group 2 (16–17 yr) n=217	Group 3 (18–19 yr) n=416	Group 4 (≥20 yr) n=145	p	Total n=878
Age, mean ± SD (yr)	14.5 ± 0.7	16.6 ± 0.5	18.5 ± 0.5	20.6 ± 1.2	—	—
BMI (kg/m²)	23.9 [21.6–26.3]	25.3 [22.1–28.4]	25.4 [22.1–28.6]	27.1 [23.9–30.3]	<0.001†	25.4 [22.2–28.6]
Menarche age (yr)	12 [12–13]	13 [12–14]	12 [11–13]	12 [11–13]	0.078	12 [12–13]
Gynaecologic age (yr)	2 [1–3]	4 [3–5]	6 [5–7]	8 [7–9]	<0.001†	6 [4–7]
Underweight (<18.5), n (%)	8 (8.0)	19 (8.8)	27 (6.5)	6 (4.1)	—	60 (6.8)
Normal weight (18.5–24.9), n (%)	57 (57.0)	81 (37.3)	170 (40.9)	42 (29.0)	—	350 (39.9)
Overweight (25.0–29.9), n (%)	30 (30.0)	77 (35.5)	141 (33.9)	57 (39.3)	—	305 (34.7)
Obese (≥30.0), n (%)	5 (5.0)	40 (18.4)	78 (18.8)	40 (27.6)	—	163 (18.6)
Overweight + obese, n (%)	35 (35.0)	117 (53.9)	219 (52.6)	97 (66.9)	<0.001‡	468 (53.3)
Clinical diagnoses at index visit, n (%)						
Oligomenorrhoea/amenorrhoea§	68 (68.0)	163 (75.1)	332 (79.8)	122 (84.1)	0.013‡	685 (78.0)
Dysmenorrhoea	14 (14.0)	50 (23.0)	100 (24.0)	29 (20.0)	0.157‡	193 (22.0)
Hirsutism (mFG ≥8)	8 (8.0)	35 (16.1)	56 (13.5)	15 (10.3)	0.166‡	114 (13.0)
Pelvic pain	12 (12.0)	39 (18.0)	75 (18.0)	23 (15.9)	0.500‡	149 (17.0)
17-OHP ≥2.0 ng/mL	0 (0.0)	4 (1.8)	7 (1.7)	5 (3.4)	0.257‡	16 (1.8)

Values are median [Q1–Q3] or n (%) as appropriate. † Kruskal–Wallis test; ‡ Chi-square test. § Oligomenorrhoea/amenorrhoea: composite of oligomenorrhoea (cycle >35 days per 2023 ESHRE criteria) and secondary amenorrhoea (≥90 days without menses). Group 4 includes patients aged 20–26 years (≥20 yr inclusion criterion; mean ± SD 20.6 ± 1.2 yr). mFG, Modified Ferriman-Gallwey.

BMI was calculated from weight and height recorded at the index visit using calibrated clinic equipment under standardised conditions. BMI was categorised using WHO adult thresholds: underweight <18.5, normal weight 18.5–24.9, overweight 25.0–29.9, and obese ≥30.0 kg/m². For patients aged <18 years (Groups 1 and 2; n=317), adult WHO BMI thresholds were applied as a pragmatic simplification compatible with retrospective HIS-derived data; although WHO 2007 age- and sex-specific BMI-for-age z-scores are the internationally preferred reference for this age range and may yield different category assignments (an effect most pronounced in Group 1), all principal analyses — including BMI–hormone Spearman correlations and multivariable logistic regression — used BMI as a continuous variable, thereby minimising dependence on categorical threshold selection. Categorical BMI data are therefore presented for descriptive purposes only and used in the WHO 2007 sensitivity analysis described below. A pre-specified sensitivity analysis was performed to address potential bias from the use of adult WHO BMI thresholds in participants aged <18 years. For this analysis, WHO 2007 age- and sex-specific BMI-for-age z-scores were calculated for participants in Groups 1 and 2 (aged 10–17 years; n=317) using the published LMS parameters, assigning each participant the mid-year age-in-months value (e.g., age 15 years = 186 months). WHO 2007 categorical thresholds were applied (thinness: z < −2 SD; overweight: z > +1 SD; obesity: z > +2 SD). For participants aged ≥18 years (Groups 3 and 4; n=561), adult WHO thresholds were retained, as our primary aim in this sensitivity analysis was to address the specific concern that adult thresholds may misclassify participants aged <18 years.

Clinical diagnoses were extracted from HIS records. Oligomenorrhoea (menstrual interval >35 days per 2023 ESHRE criteria, as documented by the attending physician) and secondary amenorrhoea (absence of menses ≥90 days) were combined into the composite variable ‘oligomenorrhoea/amenorrhoea’ because some patients were transitioning between states at diagnosis. Dysmenorrhoea was recorded as a presenting complaint based on patient-reported symptoms without formal scoring. Hirsutism was defined as a Modified Ferriman-Gallwey (mFG) score ≥8, assessed by the attending clinician at the index visit under a standardised clinic protocol; inter-rater reliability was not formally assessed (19).

All blood samples were collected on menstrual cycle days 2–5 (early follicular phase). In patients with regular cycles, sampling coincided with spontaneous menstruation. In those with oligomenorrhoea or secondary amenorrhoea, a progesterone-withdrawal bleed was induced [dydrogesterone (Duphaston) or medroxyprogesterone acetate (Tarlusal), clinician’s choice], and venepuncture performed on days 2–5 of the withdrawal bleed, ensuring basal hormonal concentrations throughout.

Laboratory parameters included FSH (IU/L), LH (IU/L), LH/FSH ratio, oestradiol (ng/L), prolactin (µg/L), TSH (mIU/L), fT4 (ng/dL), total testosterone (ng/dL), free testosterone (pg/mL), DHEA-S (µg/dL), 17-OHP (ng/mL), AMH (ng/mL), and a complete blood count. AMH, total testosterone, free testosterone, DHEA-S, 17-OHP, FSH, LH, oestradiol, prolactin, TSH, and fT4 were all measured on a Roche Cobas e-series automated analyser using electrochemiluminescence immunoassay (ECLIA; Elecsys reagents; Roche Diagnostics, Mannheim, Germany) in the ISO 15189-accredited institutional laboratory. Specifically, AMH was measured with the Elecsys AMH assay, and total testosterone with the Elecsys Testosterone II assay. Free testosterone was measured directly by automated immunoassay rather than calculated from total testosterone and SHBG; SHBG was not consistently available across the cohort and was therefore not included in the primary analyses to maintain consistent denominators, and the limitations of direct free testosterone immunoassays in adolescents are addressed in the Discussion. Total testosterone was measured by immunoassay rather than by LC-MS/MS, the 2023 ESHRE reference standard for low-range testosterone, so the absolute testosterone values and thresholds reported here should be interpreted with corresponding caution (20, 21).

The distribution of each continuous variable was assessed with the Shapiro–Wilk test; because the primary hormonal variables departed from normality, non-parametric methods were used throughout. Primary outcomes were between-group differences in LH/FSH ratio, AMH, total testosterone, and DHEA-S across age subgroups, quantified by Kruskal–Wallis H and ϵ² effect sizes. Secondary/exploratory outcomes included BMI distribution, clinical diagnosis prevalences, BMI–hormone correlations, logistic regression for hirsutism predictors, and within-PCOS empirical centile distributions. Statistical analyses were performed with SPSS 27.0 (IBM Corp., Armonk, NY) for primary analyses (Kruskal–Wallis tests, Mann–Whitney U pairwise comparisons, Spearman correlations, chi-square tests, and binary logistic regression); supplementary BMI-adjusted multivariable median (quantile) regression and log-linear regression analyses were performed with Python 3.11 (statsmodels v0.14). Continuous variables are reported as median [IQR]. Kruskal–Wallis (KW) tests with Bonferroni-corrected pairwise Mann–Whitney U tests (k=6 comparisons; Bonferroni-adjusted α=0.0083) were used for between-group comparisons; effect sizes were reported per the recommendation of Lakens (22) using epsilon-squared, calculated as ϵ²=(H−k+1)/(n−k). Categorical variables were compared with Pearson’s chi-square. Spearman’s ρ quantified monotonic associations. Logistic regression for hirsutism included three covariates (age, total testosterone, BMI; EPV = 38.0). No evidence of multicollinearity was observed among predictors (variance inflation factors: age 1.04, total testosterone 1.02, BMI 1.04; all VIF <2). Model performance was evaluated by discrimination — the area under the receiver operating characteristic curve (AUROC) with a 1,000-resample bootstrap CI, complemented by internally validated (bootstrap optimism-corrected and 10-fold cross-validated) AUROC — and by calibration, assessed with the Hosmer–Lemeshow test and its full decile table, an out-of-sample (cross-validated) calibration slope, and the Brier score; the apparent in-sample calibration slope and intercept are reported for completeness but, as mathematical identities of the maximum-likelihood fit, are not interpreted as evidence of calibration. A sensitivity logistic regression was additionally performed using biochemical hyperandrogenism (total testosterone >55 ng/dL) as the outcome, with age and BMI as covariates, to examine whether predictor associations were robust to the use of a clinician-assessed (mFG ≥8) versus a laboratory-defined hyperandrogenism phenotype. Mann–Whitney U tests compared hormonal and BMI values between patients with and without each clinical diagnosis. Empirical P5–P95 centiles were derived within each subgroup. A formal family-wise correction was not applied across the full test battery; findings with p values between 0.013 and 0.05 should be interpreted with appropriate caution. However, primary outcomes (LH/FSH ratio, AMH, total testosterone, and DHEA-S across age subgroups; total testosterone as predictor of hirsutism) were pre-specified prior to analysis, and demonstrated highly consistent patterns across multiple independent statistical approaches (Kruskal–Wallis, Bonferroni-corrected pairwise Mann–Whitney U, Spearman correlations, logistic regression) and eight pre-specified sensitivity analyses. This consistency substantially reduces the likelihood that the primary associations are attributable to type I error inflation. Two-tailed p<0.05 denoted significance. Eight pre-specified sensitivity analyses were performed: (i) repetition of all primary analyses excluding Group 1 (Groups 2–4; n=778) to address the diagnostic uncertainty in patients aged ≤15 years; (ii) reclassification of BMI categories in participants aged <18 years using WHO 2007 BMI-for-age z-scores with re-computation of BMI–hormone correlations and a parallel logistic regression for hirsutism; (iii) repetition of all primary analyses restricted to the Rotterdam-strict subset (n=830) to address potential diagnostic misclassification in patients meeting only one Rotterdam criterion; (iv) BMI-adjusted multivariable median (quantile) regression models for each primary hormonal endpoint with age subgroup as the main predictor and BMI as a continuous covariate, to formally test whether the mid-adolescent peak persisted after adjustment for the parallel BMI gradient; (v) repetition of all primary analyses restricted to an adolescent (≤19 yr) guideline-concordant subset fulfilling the 2023 ESHRE adolescent PCOS criteria (concurrent oligo-/amenorrhoea AND clinical or biochemical hyperandrogenism; n=299), to address the more stringent diagnostic threshold recommended for adolescents; (vi) repetition of all primary analyses restricted to the strict Phenotype A subset (full Rotterdam triad: oligo-/amenorrhoea + hyperandrogenism + PCOM; n=207), to test whether the mid-adolescent hormonal peak persists when phenotypically homogeneous patients are analysed in isolation; (vii) median-split temporal sensitivity analysis (early period: visits ≤2025-01-24, n=439; late period: visits >2025-01-24, n=438) to assess potential temporal heterogeneity in hormonal patterns or diagnostic patterns across the 25-month study period; and (viii) re-stratification of all primary hormonal endpoints by gynaecologic age (years post-menarche) into four strata (<2 yr, 2–3 yr, 4–6 yr, ≥7 yr post-menarche), to evaluate whether the hormonal patterns observed across chronological age subgroups also map to time since menarche as a more direct proxy for HPO-axis maturation. Throughout the manuscript, only the four pre-specified primary endpoints (LH/FSH ratio, AMH, total testosterone, and DHEA-S between-subgroup differences) and the pre-specified hirsutism logistic regression are considered confirmatory and were Bonferroni-protected; all secondary, exploratory, and *post-hoc* tests are reported as nominal p-values and should be interpreted as hypothesis-generating.

## Results

From a source population of 11,324 adolescent outpatient visits, 1,228 patients received a clinician-assigned PCOS diagnosis (10.8%), of whom 878 met all inclusion criteria after exclusion of 350 patients (**Supplementary Figure 1**). The final cohort was recruited across nine consecutive calendar quarters (range 82–130 visits per quarter). All 878 patients had complete laboratory and anthropometric panels.

Demographic, anthropometric, and clinical characteristics are summarised in **Table 1**. BMI increased significantly across subgroups (H = 25.6, p<0.001, ϵ²=0.026), rising from a median of 23.9 kg/m² in Group 1 to 27.1 kg/m² in Group 4 (Spearman ρ=+0.151, p<0.001). Despite statistical significance, this effect size was small (ϵ²=0.026), indicating limited clinical relevance and suggesting that observed differences may largely reflect physiological growth patterns rather than meaningful metabolic divergence. This gradient partly reflects normal pubertal anthropometric development in addition to any PCOS-related metabolic contribution. The proportion of overweight-or-obese patients rose from 35.0% (Group 1) to 66.9% (Group 4; χ²=24.3, p<0.001), with 53.3% overall meeting this criterion. Overall, 18.6% were obese and 6.8% underweight; the underweight proportion was not significantly associated with any hormonal parameter. Median age at menarche was 12 years overall (IQR 12–13; range 9–16; **Table 1**) and did not differ significantly across age subgroups (Kruskal–Wallis p=0.078), although Group 2 had a marginally later median menarche (13 yr) than Groups 1, 3, and 4 (12 yr each); this between-group difference was not statistically significant after correction. As expected, gynaecologic age (years since menarche) increased monotonically across age subgroups (**Table 1**), with median values of 2 years in Group 1, 4 years in Group 2, 6 years in Group 3, and 8 years in Group 4 (Kruskal–Wallis H = 524.3, p<0.001). Forty-seven patients (5.4%) were within 2 years of menarche (39 in Group 1, 8 in Group 2, none in Groups 3–4), the interval during which physiological anovulation overlaps with PCOS-like hormonal features per the 2023 ESHRE guideline; these patients were retained in the analytic cohort and addressed in a dedicated gynaecologic-age sensitivity analysis (described below; **Supplementary Table 5**). Oligomenorrhoea/amenorrhoea was the most prevalent diagnosis (78.0%; χ²=10.86, p=0.013), increasing progressively from 68.0% in Group 1 to 84.1% in Group 4. Dysmenorrhoea was present in 22.0% (p=0.157), hirsutism in 13.0% (p=0.166), and pelvic pain in 17.0% (p=0.500).

Criterion-level verification confirmed that 94.5% (830/878) of the analytic cohort met at least two of three Rotterdam criteria. Oligo-/amenorrhoea was documented in 78.0% (n=685), clinical or biochemical hyperandrogenism in 56.9% (n=500), and PCOM on ultrasound in 83.1% (n=730). Rotterdam phenotype distribution was: phenotype A (HA + oligo-anovulation + PCOM) 23.6% (n=207), phenotype B (HA + oligo-anovulation) 14.8% (n=130), phenotype C (HA + PCOM, ovulatory) 18.6% (n=163), and phenotype D (oligo-anovulation + PCOM, non-hyperandrogenic) 37.6% (n=330). The remaining 5.5% (n=48) met only one Rotterdam criterion on structured re-abstraction; of these, 68.8% (n=33) had supportive biochemical evidence of PCOS (AMH >4.5 ng/mL or LH/FSH ratio >2.0), and the remainder retained their original clinician-assigned diagnosis based on additional clinical features (acne, androgenic alopecia, family history) not systematically captured in the structured HIS dataset. Rotterdam criteria fulfilment was consistent across age subgroups (Group 1: 93.0%; Group 2: 90.8%; Group 3: 95.4%; Group 4: 98.6%); full phenotype distribution by age subgroup is presented in **Table 2**.

**Table 2:** Rotterdam criteria fulfilment and phenotype distribution by age subgroup.

Rotterdam criterion/phenotype	Group 1 (≤15 yr) n=100	Group 2 (16–17 yr) n=217	Group 3 (18–19 yr) n=416	Group 4 (≥20 yr) n=145	Total n=878
Individual criteria, n (%)					
Oligo-/amenorrhoea (C1)	—	—	—	—	685 (78.0)
Hyperandrogenism, clinical or biochemical (C2)†	—	—	—	—	500 (56.9)
PCOM on ultrasound (C3)	—	—	—	—	730 (83.1)
Rotterdam phenotypes, n (%)					
A (C1 + C2 + C3)	18 (18.0)	52 (24.0)	104 (25.0)	33 (22.8)	207 (23.6)
B (C1 + C2, PCOM–)	7 (7.0)	54 (24.9)	64 (15.4)	5 (3.4)	130 (14.8)
C (C2 + C3, ovulatory)	25 (25.0)	45 (20.7)	72 (17.3)	21 (14.5)	163 (18.6)
D (C1 + C3, non-HA)	43 (43.0)	46 (21.2)	157 (37.7)	84 (57.9)	330 (37.6)
Summary					
≥2 criteria met, n (%)	93 (93.0)	197 (90.8)	397 (95.4)	143 (98.6)	830 (94.5)
<2 criteria (single criterion)‡	7 (7.0)	20 (9.2)	19 (4.6)	2 (1.4)	48 (5.5)

† Clinical hyperandrogenism defined as mFG score ≥8; biochemical hyperandrogenism as total testosterone >55 ng/dL. ‡ Of these 48 patients, 33 (68.8%) had supportive biochemical evidence (AMH >4.5 ng/mL or LH/FSH >2.0). HA, hyperandrogenism; PCOM, polycystic ovarian morphology.

Although BMI differed significantly across age subgroups (**Table 1**), it was weakly and largely non-significantly correlated with the primary hormonal markers reported in **Tables 3**, **4**: LH/FSH ratio (ρ=−0.048, p=0.158), AMH (ρ=−0.030, p=0.369), total testosterone (ρ=+0.075, p=0.026), DHEA-S (ρ=−0.046, p=0.175), and oestradiol (ρ=+0.056, p=0.096). Only total testosterone reached nominal significance with a negligible effect. Obese patients (BMI ≥30 kg/m²) did not differ from non-obese patients in LH/FSH ratio (2.00 vs 1.98, p=0.510) or AMH (4.75 vs 4.63 ng/mL, p=0.852). BMI was not significantly associated with oligomenorrhoea/amenorrhoea (25.3 vs 25.6 kg/m², p=0.591) or hirsutism (26.6 vs 25.3 kg/m², p=0.066).

**Table 3:** Pituitary–gonadal, thyroid, and haematological parameters by age subgroup. Values are median [Q1–Q3]. ϵ², epsilon-squared effect size.

Parameter	Group 1 (≤15 yr)	Group 2 (16–17 yr)	Group 3 (18–19 yr)	Group 4 (≥20 yr)	p (KW)	ϵ²
Pituitary–gonadal hormones						
FSH (IU/L)	4.10 [3.50–5.00]	4.70 [4.00–5.40]	5.20 [4.40–6.00]	5.70 [4.90–6.40]	<0.001	0.113
LH (IU/L)	8.15 [6.40–9.50]	10.70 [9.20–12.80]	10.50 [8.88–12.20]	9.00 [7.30–11.00]	<0.001	0.112
LH/FSH ratio	1.90 [1.44–2.45]	2.33 [1.84–2.98]	2.02 [1.61–2.54]	1.60 [1.35–1.97]	<0.001	0.098
LH/FSH >2.0, n (%)	44 (44.0)	144 (66.4)	209 (50.2)	35 (24.1)	<0.001*	—
Oestradiol (ng/L)	34.0 [21.0–47.0]	53.0 [42.0–64.0]	70.0 [58.8–83.0]	81.0 [71.0–94.0]	<0.001	0.390
Prolactin (µg/L)	13.1 [10.5–16.5]	13.7 [10.6–16.9]	14.4 [10.9–17.9]	15.0 [11.9–17.6]	0.080	0.004
Thyroid function						
TSH (mIU/L)	2.25 [1.71–2.77]	2.20 [1.64–2.67]	2.25 [1.60–2.79]	2.19 [1.59–2.91]	0.903	0.000
fT4 (ng/dL)	1.13 [1.02–1.25]	1.16 [1.01–1.28]	1.14 [1.02–1.29]	1.11 [0.99–1.22]	0.079	0.004
Complete blood count						
Haemoglobin (g/dL)	13.3 [12.6–13.8]	13.2 [12.5–13.9]	13.3 [12.7–13.8]	13.3 [12.7–13.9]	0.928	0.000
Hb <12.0 g/dL, n (%)	2 (2.0)	22 (10.1)	31 (7.5)	15 (10.3)	0.057*	—
MCV (fL)	87.0 [84.0–91.0]	86.0 [83.0–90.0]	87.0 [84.0–91.0]	86.0 [83.0–90.0]	0.117	0.003
MCV <80 fL, n (%)	3 (3.0)	23 (10.6)	24 (5.8)	7 (4.8)	0.029*	—
RDW-CV (%)	13.2 [12.5–13.8]	13.3 [12.4–13.9]	13.3 [12.4–14.1]	13.3 [12.5–14.0]	0.788	0.000

*Chi-square test. KW, Kruskal–Wallis. ϵ²: <0.06 small, 0.06–0.14 medium, >0.14 large.

**Table 4:** Androgen panel, AMH, and post-hoc pairwise comparisons by age subgroup.

Parameter	Group 1 (≤15 yr)	Group 2 (16–17 yr)	Group 3 (18–19 yr)	Group 4 (≥20 yr)	p (KW)	ϵ²
Total testosterone (ng/dL)	55.1 [39.9–66.8]	64.5 [52.9–77.1]	58.5 [46.3–70.4]	49.1 [37.5–62.1]	<0.001	0.064
Free testosterone (pg/mL)	0.86 [0.68–1.17]	1.18 [0.92–1.52]	1.08 [0.82–1.38]	0.92 [0.65–1.21]	<0.001	0.050
DHEA-S (µg/dL)	290.5 [246.5–335.0]	311.0 [274.0–364.0]	277.0 [226.8–322.5]	251.0 [199.0–292.0]	<0.001	0.094
17-OHP (ng/mL)	0.88 [0.64–1.05]	0.84 [0.60–1.07]	0.85 [0.68–1.05]	0.81 [0.65–1.05]	0.794	0.000
AMH (ng/mL)	3.61 [2.54–4.38]	5.31 [4.36–6.57]	4.99 [4.00–6.04]	3.39 [2.52–4.41]	<0.001	0.203
AMH >4.5 ng/mL, n (%)	23 (23.0)	152 (70.0)	261 (62.7)	35 (24.1)	<0.001^¶^	—
AMH >7.0 ng/mL, n (%)	2 (2.0)	39 (18.0)	37 (8.9)	0 (0.0)	<0.001*	—
Post-hoc pairwise comparisons (Mann–Whitney U, Bonferroni-corrected; adjusted α=0.0083)						
Comparison	LH/FSH		AMH	Total-T		DHEA-S
Group 1 vs Group 2	<0.001**		<0.001**	<0.001**		0.033 ns
Group 1 vs Group 3	0.973 ns		<0.001**	0.067 ns		0.277 ns
Group 1 vs Group 4	0.004**		1.000 ns	0.992 ns		<0.001**
Group 2 vs Group 3	<0.001**		0.009 ns	0.002**		<0.001**
Group 2 vs Group 4	<0.001**		<0.001**	<0.001**		<0.001**
Group 3 vs Group 4	<0.001**		<0.001**	<0.001**		0.001**

Values are median [Q1–Q3] unless stated. ^¶^Chi-square test. ϵ², epsilon-squared. Bonferroni-corrected pairwise p-values: **Bonferroni-adjusted p<0.0083 (k=6 comparisons, α=0.0083); *Bonferroni-adjusted p<0.05; ns, not significant.

A pre-specified sensitivity analysis reclassifying participants aged <18 years using WHO 2007 BMI-for-age z-scores (**Supplementary Table 1**) yielded higher overweight-plus-obesity prevalences than adult thresholds: 62.0% in Group 1 (vs 35.0%) and 61.8% in Group 2 (vs 53.9%). The overall cohort prevalence rose from 53.3% to 58.3% (χ²=11.5, p=0.009 across age subgroups). Despite this reclassification, the principal findings were preserved: (i) Spearman correlations between WHO z-score and primary hormonal markers in Groups 1 and 2 (n=317) remained weak and non-significant (LH/FSH ratio ρ=−0.091, p=0.107; AMH ρ=−0.035, p=0.535; total testosterone ρ=+0.086, p=0.125; DHEA-S ρ=+0.019, p=0.733); (ii) in a parallel logistic regression restricted to participants aged <18 years (n=317, events=43) with WHO z-score substituted for BMI, total testosterone remained the sole significant predictor of hirsutism (OR 1.057, 95% CI 1.034–1.081, p<0.001), whereas WHO z-score was non-significant (OR 1.065, p=0.589). These results confirm that the principal conclusions of the study are robust to BMI classification methodology.

In an additional sensitivity analysis restricted to the subset of patients strictly fulfilling ≥2 Rotterdam criteria (n=830; excluding 48 patients with single-criterion diagnosis), all primary findings were preserved. The mid-adolescent peak remained significant for LH/FSH ratio (ϵ²=0.092), AMH (ϵ²=0.201), total testosterone (ϵ²=0.081), and DHEA-S (ϵ²=0.085; all p<0.001) — closely matching the full-cohort effect sizes reported in **Table 4**. BMI–hormone correlations remained weak and largely non-significant (all |ρ|<0.09). In parallel logistic regression (n=830, events=114, EPV = 38.0, McFadden R²=0.160), total testosterone remained the sole significant predictor of hirsutism (OR 1.067, 95% CI 1.052–1.083, p<0.001), with age and BMI non-significant (p=0.656 and p=0.266, respectively), again concordant with the primary model in **Table 5**. These results confirm that the principal conclusions were not driven by potential diagnostic misclassification in the subset of patients meeting only one Rotterdam criterion.

**Table 5:** Logistic regression model for hirsutism (n=878, events=114, EPV = 38.0, McFadden R²=0.172).

Predictor	β	SE	OR	95% CI	p
Age (yr)	0.034	0.066	1.035	0.910–1.177	0.605
Total testosterone (ng/dL)	0.068	0.007	1.070	1.055–1.086	<0.001
BMI (kg/m²)	0.024	0.022	1.024	0.980–1.069	0.291

OR, odds ratio; SE, standard error; EPV, events per variable. Covariates: age, total testosterone, BMI. Model performance: apparent AUROC 0.818 (95% CI 0.785–0.850), optimism-corrected 0.815, 10-fold cross-validated 0.813; Brier score 0.102 (null 0.113); out-of-sample calibration slope 0.965. The apparent in-sample calibration slope (1.00) and intercept (0.00) are mathematical identities of the maximum-likelihood fit; the significant Hosmer–Lemeshow test (χ²=49.8, df=8, p<0.001) reflects near-complete separation on total testosterone (see Results and **Table 7**).

In BMI-adjusted multivariable median (quantile) regression models (**Supplementary Table 2**), the mid-adolescent peak (Group 2 vs Group 1, reference) remained significant after adjustment for BMI as a continuous covariate for each primary hormonal endpoint: LH/FSH ratio (β=+0.40, p<0.001; BMI p=1.000), AMH (β=+1.72, p<0.001; BMI p=0.679), total testosterone (β=+8.24 ng/dL, p=0.004; BMI β=+0.43, p=0.011), and DHEA-S (β=+20.81 µg/dL, p=0.049; BMI p=0.518). BMI itself reached statistical significance only in the total testosterone model and with a small effect size; coefficient changes for the Group 2 effect after BMI adjustment were negligible across all four endpoints (range −13.2% to +3.6%). Parallel log-linear regression yielded concordant results (**Supplementary Table 2** footnote), with Group 2 ratios of 1.26 (LH/FSH), 1.61 (AMH), 1.24 (total testosterone), and 1.08 (DHEA-S) versus Group 1, all p<0.05; BMI was not significant for LH/FSH, AMH, or DHEA-S (all p>0.4). These models support that the mid-adolescent hormonal pattern was not materially explained by BMI as measured in this cohort.

In a final sensitivity analysis restricted to adolescents (≤19 yr) fulfilling the 2023 ESHRE adolescent PCOS criteria (n=299; concurrent oligo-/amenorrhoea and clinical or biochemical hyperandrogenism), the mid-adolescent peak was preserved across all primary hormonal markers, although with somewhat smaller effect sizes than in the full cohort (**Table 4** for full-cohort comparators): LH/FSH ratio (Group 1: 2.10; Group 2: 2.40; Group 3: 2.00; ϵ²=0.039, p=0.001), AMH (4.00; 5.35; 5.15 ng/mL; ϵ²=0.087, p<0.001), total testosterone (62.9; 68.7; 65.5 ng/dL; ϵ²=0.026, p=0.008), and DHEA-S (298.0; 304.0; 272.0 µg/dL; ϵ²=0.069, p<0.001). BMI–hormone correlations remained negligible in this subset (all |ρ|<0.11). In parallel logistic regression (n=299, events=58, McFadden R²=0.059), total testosterone remained the sole significant predictor of hirsutism (OR 1.054, 95% CI 1.027–1.082, p<0.001), with age and BMI non-significant. These results indicate that the principal conclusions of the study — the mid-adolescent peak not materially explained by BMI in adjusted models and the role of total testosterone as the strongest available biochemical correlate of recorded hirsutism — are robust within the more stringent adolescent guideline-concordant subgroup. Of note, only 299/733 (40.8%) of patients aged ≤19 years fulfilled the strict 2023 ESHRE adolescent PCOS criteria, highlighting that the original clinician-assigned cohort included a substantial proportion of patients meeting Rotterdam criteria but not the more stringent adolescent diagnostic threshold; the preservation of all principal findings in this stricter subset nonetheless supports the robustness of the main conclusions.

Because Phenotype D (oligo-anovulation with polycystic ovarian morphology, without hyperandrogenism) was the largest phenotype (37.6%, n=330; Group 1 n=43, Group 2 n=46, Group 3 n=157, Group 4 n=84), the primary age–hormone pattern was examined within this subset as a main-body secondary analysis. The higher mid-adolescent AMH values were preserved (Group 2 median 5.05 vs Group 1 3.5 ng/mL; ϵ²=0.197, p<0.001), closely matching the full-cohort effect (ϵ²=0.203); the LH/FSH gradient was also preserved (ϵ²=0.108), whereas the DHEA-S gradient was attenuated (ϵ²=0.028). Because Phenotype D is defined without any androgen criterion, the persistence of the AMH pattern within it indicates that the mid-adolescent AMH signal is not an artefact of hyperandrogenism-based case selection, complementing the strict Phenotype A analysis (23).

A pre-specified sensitivity analysis restricted to the strict Phenotype A subset (full Rotterdam triad: oligo-/amenorrhoea + hyperandrogenism + PCOM; n=207; Group 1 n=18, Group 2 n=52, Group 3 n=104, Group 4 n=33) yielded heterogeneous results across the four primary endpoints (**Supplementary Table 3**). The AMH peak was almost entirely preserved (Group 2 median 5.70 ng/mL vs Group 1 3.95; ϵ²=0.193, p<0.001), with effect size attenuation of only 5% relative to the full cohort (full cohort ϵ²=0.203). The DHEA-S age-gradient remained statistically significant overall (ϵ²=0.072, p<0.001), but the Group 2 peak observed in the full cohort was not preserved within Phenotype A: Group 1 and Group 2 medians were closely comparable (298.5 µg/dL and 291.0 µg/dL, respectively), with the gradient driven principally by the lower values in Groups 3 (264.5) and 4 (241.0). For LH/FSH ratio and total testosterone, ϵ² values were attenuated by approximately 50% within Phenotype A (LH/FSH 0.098 → 0.043; total testosterone 0.064 → 0.028), although Group 2 retained the highest median value for both endpoints (LH/FSH 2.25; total testosterone 64.2 ng/dL). The total testosterone attenuation is partially attributable to a definitional artefact: Phenotype A requires fulfilment of the hyperandrogenism criterion (C2; total testosterone >55 ng/dL or modified Ferriman–Gallwey ≥8), and within the Group 1 Phenotype A subset (n=18), all 18 patients met C2 via biochemical hyperandrogenism (total testosterone >55 ng/dL), with none meeting the criterion via clinical hirsutism alone. This biochemical-floor selection raised the Group 1 Phenotype A median total testosterone from 55.1 ng/dL (full cohort Group 1) to 60.2 ng/dL (Phenotype A Group 1), thereby narrowing the Group 2–Group 1 contrast. The robustness of the AMH peak — which is not part of the Rotterdam definition and therefore unaffected by selection on hyperandrogenism — within the strict Phenotype A subset is the strongest single-endpoint support for an HPO-axis maturation interpretation rather than diagnostic-stage selection bias.

A median-split temporal sensitivity analysis (early period: visits ≤2025-01-24, n=439; late period: visits >2025-01-24, n=438; one patient with missing visit date excluded) showed no meaningful temporal heterogeneity (**Supplementary Table 4**). Median values for all four primary endpoints were stable between halves (LH/FSH 1.90 vs 2.00, p=0.23; AMH 4.60 vs 4.80 ng/mL, p=0.11; total testosterone 58.3 vs 58.4 ng/dL, p=0.66; DHEA-S 278.0 vs 287.5 µg/dL, p=0.02), as was BMI (25.9 vs 25.3 kg/m², p=0.16). The mid-adolescent peak was preserved within both halves for LH/FSH ratio (early ϵ²=0.119; late ϵ²=0.067), AMH (early ϵ²=0.217; late ϵ²=0.180), total testosterone (early ϵ²=0.076; late ϵ²=0.050), and DHEA-S (early ϵ²=0.115; late ϵ²=0.067; Group 2 median 314.0 vs Group 1 284.0 in early half; Group 1 302.0 vs Group 2 299.5 in late half — a 0.8% difference of negligible clinical significance). Outcome prevalences were comparable between halves (hirsutism 11.2% vs 14.8%, p=0.13; oligomenorrhoea/amenorrhoea 80.0% vs 76.0%, p=0.19). These results indicate that no diagnostic, laboratory, or population shifts during the 25-month study period materially affected the principal findings.

A pre-specified gynaecologic-age sensitivity analysis re-stratified all 878 patients into four post-menarchal strata (<2 yr, n=47; 2–3 yr, n=136; 4–6 yr, n=384; ≥7 yr, n=311; **Supplementary Table 5**). All four primary hormonal endpoints showed statistically significant variation across gynaecologic-age strata (LH/FSH ϵ²=0.044, p<0.001; AMH ϵ²=0.055, p<0.001; total testosterone ϵ²=0.011, p=0.007; DHEA-S ϵ²=0.027, p<0.001), with the highest median values observed in the 2–3 yr post-menarche stratum for LH/FSH ratio (2.20) and total testosterone (62.2 ng/dL), and in the 4–6 yr stratum for AMH (5.00 ng/mL) and DHEA-S (293.5 µg/dL). The 47 patients within 2 years of menarche, in whom physiological anovulation overlaps with PCOS-like features, showed lower median values for all four endpoints than the 2–3 yr stratum, supporting the 2023 ESHRE caution against early adolescent diagnostic application of these markers. Effect sizes were attenuated relative to chronological-age stratification (e.g., AMH ϵ² 0.203 → 0.055), and Spearman correlations of the four primary hormones with gynaecologic age were weaker than with chronological age (e.g., AMH ρ=−0.119 vs −0.154; DHEA-S ρ=−0.188 vs −0.253). In multivariable median regression including chronological age, age at menarche, and BMI as joint predictors, chronological age remained an independent significant predictor for all four endpoints, whereas age at menarche was non-significant in every model (all p>0.25), indicating that chronological age captures the dominant signal in this cohort, with menarchal timing not adding independent explanatory variance.

**Figure 1:**
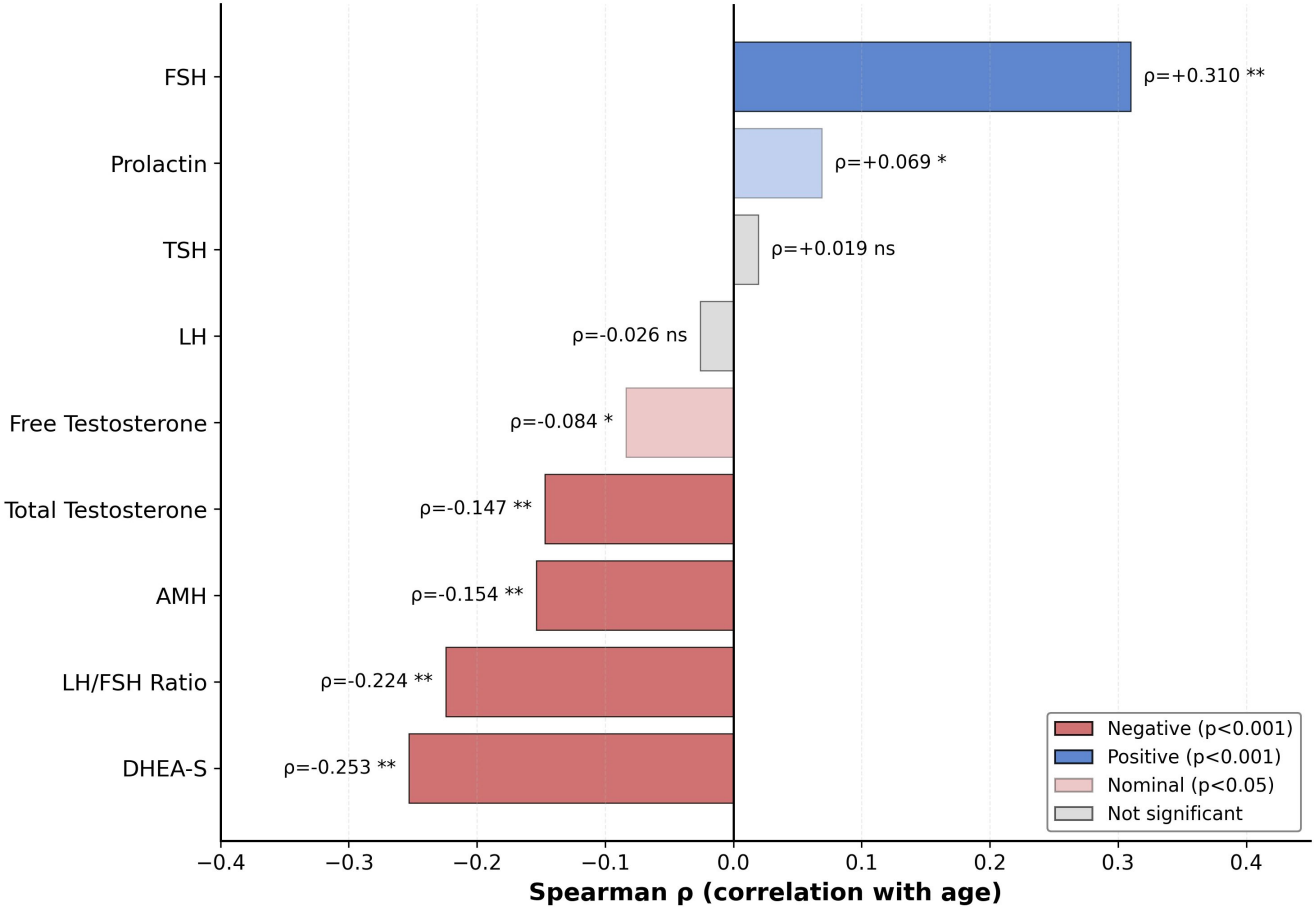
Spearman rank correlation coefficients between age and key hormonal parameters (n=878). Dark red bars, significant negative correlations (p<0.001); dark blue bars, significant positive correlations (p<0.001); pale bars, nominal significance (p<0.05); grey bars, not significant. **p<0.001; *p<0.05; ns, not significant.

**Figure 2:**
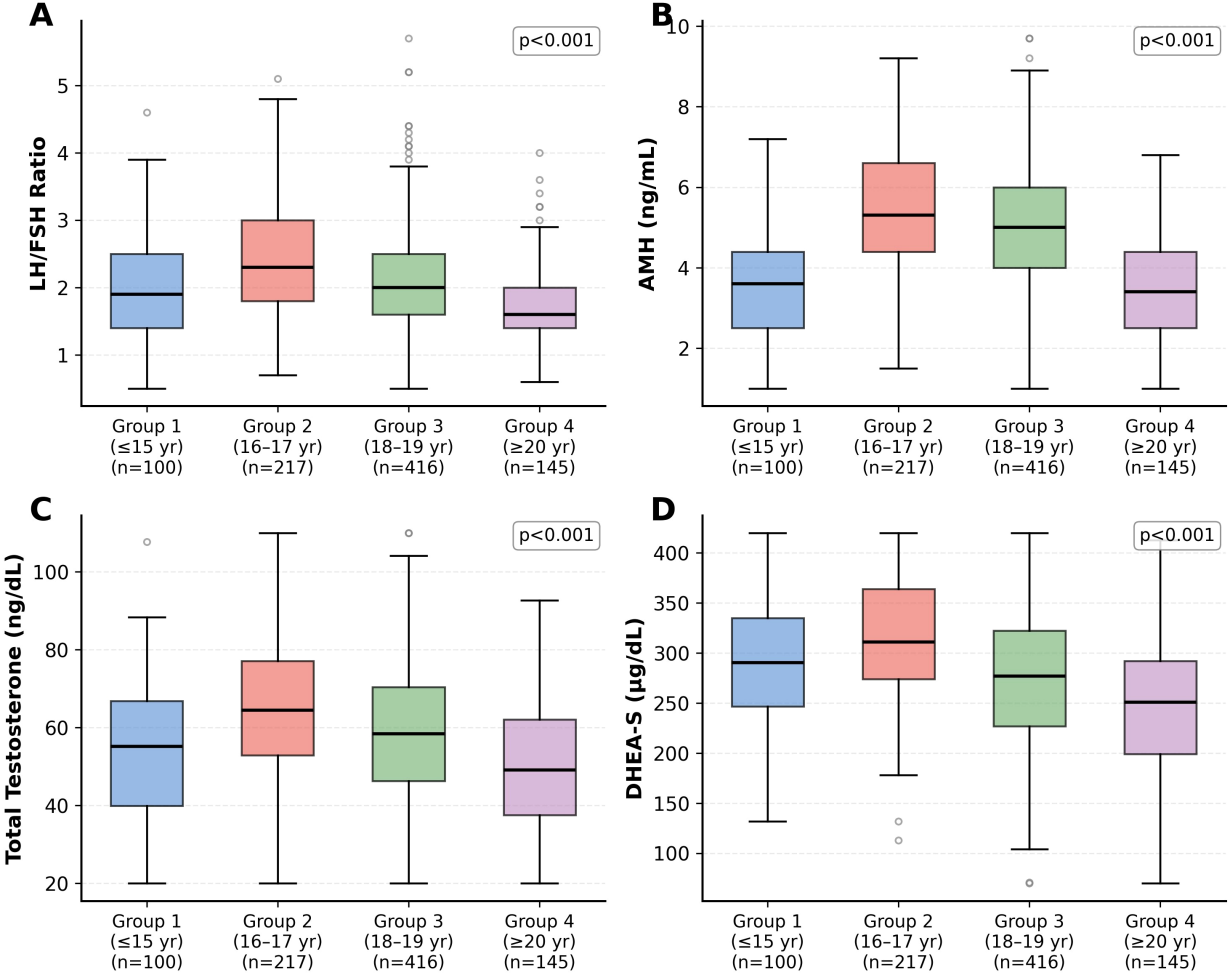
Distribution of primary hormonal markers across four age subgroups (n=878). Panel **(A)** LH/FSH ratio (ϵ²=0.098). Panel **(B)** AMH, ng/mL (ϵ²=0.203). Panel **(C)** total testosterone, ng/dL (ϵ²=0.064). Panel **(D)** DHEA-S, µg/dL (ϵ²=0.094). Boxes, IQR; horizontal lines, median; whiskers, 1.5×IQR; circles, outliers. Kruskal–Wallis p<0.001 for all panels, with the highest median values in Group 2 (mid-adolescent) for each endpoint and lower values in older subgroups; the AMH peak was the most robust across BMI-adjusted and phenotype-restricted sensitivity analyses (see Results).

Pituitary–gonadal, thyroid, and haematological parameters are presented in **Table 3**. FSH rose monotonically with age (ϵ²=0.113, ρ=+0.310, p<0.001; **Figure 1**). The LH/FSH ratio was highest in Group 2 (2.33 [1.84–2.98]) and lowest in Group 4 (1.60 [1.35–1.97]; ϵ²=0.098, p<0.001; **Figure 2A**). Oestradiol showed the largest effect size in the dataset (ϵ²=0.390, ρ=+0.569). Prolactin showed a borderline positive age correlation (ρ=+0.069, p=0.042, ϵ²=0.004; negligible effect). TSH and fT4 were stable across all subgroups (p=0.903 and p=0.079; ϵ²≤0.004). All haematological parameters showed negligible effect sizes (ϵ²≤0.003).

**Figure 3:**
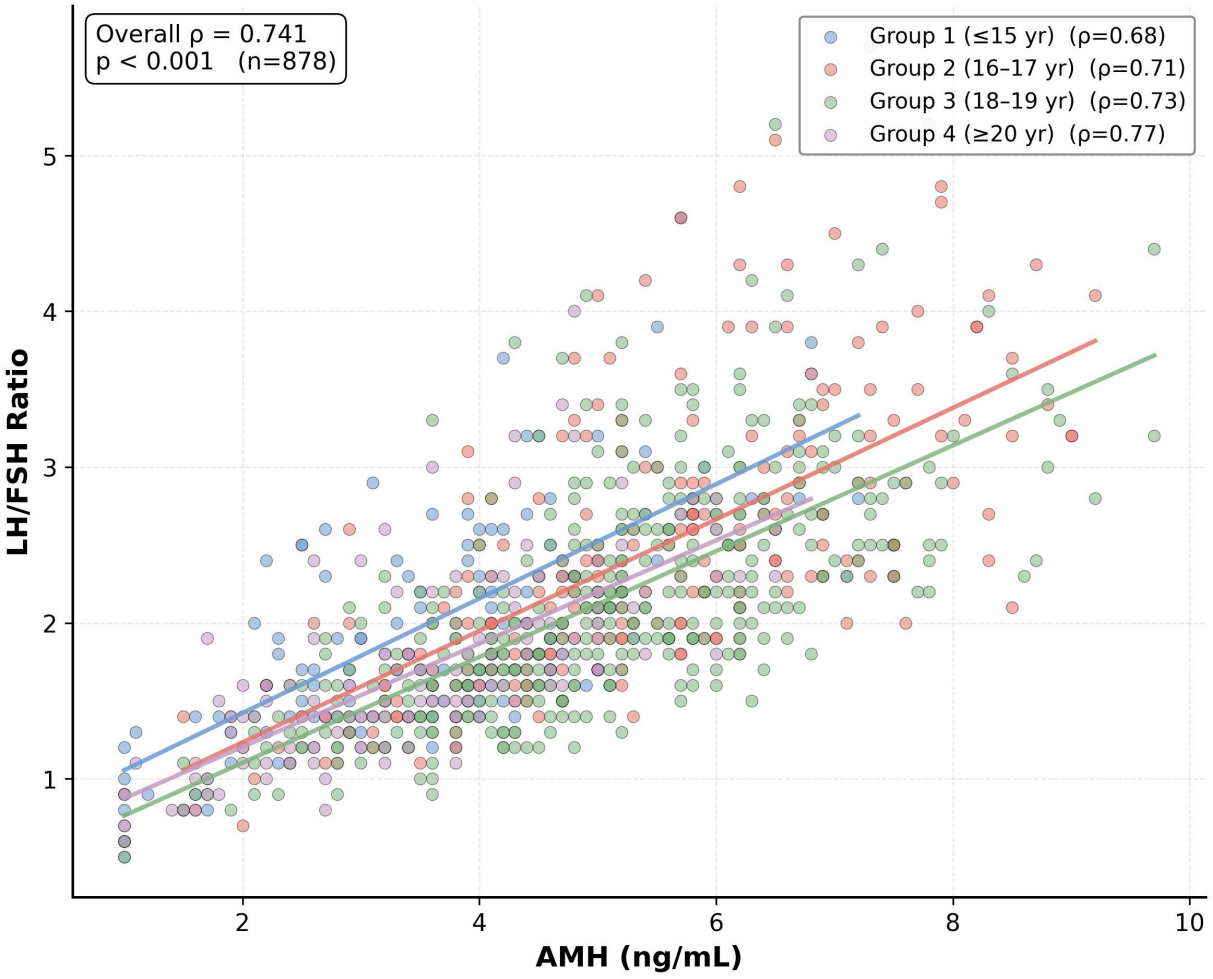
Scatter plot of AMH (ng/mL) versus LH/FSH ratio across all 878 patients, colour-coded by age subgroup. Overall Spearman ρ=0.741, p<0.001. Per-group ρ: Group 1, 0.678; Group 2, 0.709; Group 3, 0.734; Group 4, 0.773 (all p<0.001).

Androgen and AMH results, with *post-hoc* comparisons, are presented in **Table 4**. Total testosterone was highest in Group 2 (mid-adolescents) (64.5 [52.9–77.1] ng/dL; ϵ²=0.064, ρ=−0.147; **Figure 2C**). DHEA-S showed a parallel mid-adolescent maximum (ϵ²=0.094, ρ=−0.253; **Figures 1**, **2D**). AMH demonstrated the largest age-related effect size among the primary hormonal endpoints (ϵ²=0.203), with the highest median values in Groups 2 and 3 (5.31 and 4.99 ng/mL) and a markedly lower median in Group 4 (3.39 ng/mL; **Figure 2B**). AMH >4.5 ng/mL was present in 70.0% of Group 2 versus 23.0% of Group 1 (p<0.001). *Post-hoc* testing confirmed that Group 2 differed significantly from Group 4 for all four primary endpoints, and from Group 1 for LH/FSH ratio, AMH, and total testosterone; the Group 1–Group 2 difference in DHEA-S was nominal (p=0.033) but did not survive Bonferroni correction. Group 2 versus Group 3 reached significance for LH/FSH ratio, total testosterone, and DHEA-S, while the AMH difference between Groups 2 and 3 did not survive Bonferroni correction (**Table 4**). The AMH–LH/FSH correlation was strong overall (ρ=+0.741, p<0.001) and replicated within each subgroup independently (ρ=0.678–0.773; all p<0.001; **Figure 3**).

Empirical within-PCOS centile distributions (P5–P95) for the primary androgen and gonadotropin markers are presented in **Table 6**. The P95 for LH/FSH ratio in Group 2 (4.10) exceeds that of Group 4 (2.87) by 43%. Median AMH in Group 2 (5.31 ng/mL) is 47% higher than in Group 1 (3.61 ng/mL) and 57% higher than in Group 4 (3.39 ng/mL) — differences not materially explained by BMI in adjusted analyses.

**Table 6:** Empirical within-PCOS centile distributions (P5–P95) for LH/FSH ratio, AMH, and total testosterone by age subgroup.

Parameter/Group	P5	P10	P25	P50	P75	P90	P95	n
LH/FSH ratio								
Group 1 (≤15 yr)	0.93	1.19	1.44	1.90	2.45	2.84	3.20	100
Group 2 (16–17 yr)	1.31	1.51	1.84	2.33	2.98	3.71	4.10	217
Group 3 (18–19 yr)	1.16	1.30	1.61	2.02	2.54	3.09	3.44	416
Group 4 (≥20 yr)	0.88	1.03	1.35	1.60	1.97	2.50	2.87	145
AMH (ng/mL)								
Group 1 (≤15 yr)	1.04	1.70	2.54	3.61	4.38	5.37	5.93	100
Group 2 (16–17 yr)	2.84	3.41	4.36	5.31	6.57	7.55	8.25	217
Group 3 (18–19 yr)	2.53	3.13	4.00	4.99	6.04	6.88	7.49	416
Group 4 (≥20 yr)	1.53	1.76	2.52	3.39	4.41	5.16	5.91	145
Total testosterone (ng/dL)								
Group 1 (≤15 yr)	20.0	24.8	39.9	55.1	66.8	75.2	79.2	100
Group 2 (16–17 yr)	35.3	43.2	52.9	64.5	77.1	85.9	91.8	217
Group 3 (18–19 yr)	30.5	35.9	46.3	58.5	70.4	81.6	88.3	416
Group 4 (≥20 yr)	23.9	26.4	37.5	49.1	62.1	71.7	75.7	145

These distributions reflect intra-cohort variation and are not population normative reference intervals.

Values derived from non-parametric empirical distribution within each subgroup.

Oligomenorrhoea/amenorrhoea was associated with marginally higher LH/FSH ratio (2.00 vs 1.90, p=0.053) and AMH (4.70 vs 4.40 ng/mL, p=0.029), but with paradoxically lower total testosterone (54.9 vs 72.3 ng/dL, p<0.001) and DHEA-S (277.0 vs 320.0 µg/dL, p<0.001), indicating that the menstrual irregularity component is dissociated from biochemical hyperandrogenism in this cohort. Hirsutism was associated with higher total testosterone (71.8 vs 55.2 ng/dL, p<0.001) but not DHEA-S (p=0.833), supporting ovarian androgen dominance. Dysmenorrhoea and pelvic pain showed no significant hormonal associations (all p>0.26). In the logistic regression model for hirsutism (**Table 5**; n=878, events=114, EPV = 38.0, McFadden R²=0.172), total testosterone was the sole significant predictor (OR 1.07 per ng/dL, 95% CI 1.055–1.086, p<0.001; equivalent to an approximately two-fold increase per 10 ng/dL) after adjustment for age and BMI. Because SHBG and free androgen index were unavailable, total testosterone represents the strongest correlate of hirsutism within the available biochemical panel rather than a complete assessment of androgen bioavailability. BMI was not independently associated (OR 1.024, p=0.291) and age was also non-significant (OR 1.035, p=0.605). In the sensitivity analysis excluding Group 1 (n=778), all primary findings were preserved: the mid-adolescent hormonal peak remained significant for LH/FSH ratio (ϵ²=0.110), total testosterone (ϵ²=0.066), DHEA-S (ϵ²=0.105), and AMH (ϵ²=0.163; all p<0.001; G2–G3 pairwise p_adj=0.009, ns after Bonferroni correction); total testosterone remained the sole predictor of hirsutism (OR 1.072, 95% CI 1.055–1.089, p<0.001, McFadden R²=0.177). Discrimination of the primary hirsutism logistic model was good and internally robust (apparent AUROC 0.818, bootstrap 95% CI 0.785–0.850; bootstrap optimism-corrected AUROC 0.815; 10-fold cross-validated AUROC 0.813), with a Brier score of 0.102 (null 0.113). Because all 114 patients with hirsutism had total testosterone >55 ng/dL, testosterone separated cases from non-cases almost completely and predicted probabilities were concentrated at the extremes; the apparent in-sample calibration slope (1.00) and intercept (0.00) are mathematical properties of the maximum-likelihood fit and are not, by themselves, evidence of calibration, whereas the out-of-sample (cross-validated) calibration slope was 0.965. The Hosmer–Lemeshow test was significant (χ²=49.8, df=8, p<0.001); the full decile table (**Table 7**) shows that no events occurred in the lowest five deciles, the model under-predicted risk in deciles 6–8 (e.g., decile 8: 27 observed vs 15.7 expected) and over-predicted in the highest decile (28 observed vs 38.6 expected). This pattern reflects the near-complete separation on testosterone rather than a globally miscalibrated model, and indicates that the model is best interpreted as identifying total testosterone as the dominant independent correlate of hirsutism rather than as a calibrated absolute-risk tool. In a parallel sensitivity logistic regression using biochemical hyperandrogenism (total testosterone >55 ng/dL; n=500 events, 56.9%) as the outcome with age and BMI as covariates, age was a significant inverse predictor (OR 0.896 per year, 95% CI 0.833–0.964, p=0.003), whereas BMI was non-significant (OR 1.022 per kg/m², 95% CI 0.994–1.051, p=0.132). The 100% concordance of clinical hirsutism (mFG ≥8) with biochemical hyperandrogenism (all 114 hirsutism patients had total testosterone >55 ng/dL) supports the diagnostic coherence of the two definitions, while the negative age coefficient is consistent with the mid-adolescent hormonal peak.

**Table 7:** Hosmer–Lemeshow calibration table for the hirsutism logistic model (n=878, 114 events): observed and expected events across deciles of predicted risk (χ²=49.8, df=8, p<0.001).

Decile	n	Observed	Expected	Obs. %	Exp. %
1	88	0	0.9	0.0	1.1
2	88	0	2.1	0.0	2.4
3	88	0	3.3	0.0	3.8
4	87	0	4.8	0.0	5.5
5	88	0	6.4	0.0	7.3
6	88	15	8.6	17.0	9.8
7	87	22	11.6	25.3	13.3
8	88	27	15.7	30.7	17.8
9	88	22	22.0	25.0	25.0
10	88	28	38.6	31.8	43.9

## Discussion

This study provides one of the largest age-stratified characterisations of hormonal, anthropometric, and clinical profiles in a Turkish adolescent and young adult clinician-assigned PCOS cohort (n=878), drawn from 1,228 PCOS-diagnosed patients across 11,324 consecutive adolescent outpatient visits (clinic-based diagnosis rate of 10.8%). Adolescent PCOS represents a particular diagnostic and management challenge owing to substantial overlap with normal pubertal physiology (24, 25). Three key findings emerge from the present cohort. First, BMI increased substantially across age subgroups yet was weakly and largely non-significantly correlated with the primary hormonal markers. Second, a mid-adolescent enrichment was observed across multiple hormonal markers despite this BMI gradient, with the AMH peak being the most robust signal across phenotype-restricted and BMI-adjusted analyses. Third, total testosterone predicted hirsutism independently of BMI within the available biochemical panel.

That BMI did not significantly predict the primary hormonal variation is clinically informative. In adult PCOS, obesity is a well-established modulator of AMH and androgens, with most published series reporting inverse BMI–AMH and positive BMI–androgen correlations (8, 16, 26). In our cohort, these associations were either non-significant or negligible in effect size. We propose two non-exclusive explanations. First, the cohort’s BMI range [median 25.4 kg/m² (IQR 22.2–28.6)] may be insufficient to detect BMI-dependent hormonal variation across the full adiposity spectrum; only 18.6% met the criteria for obesity. Second, HPO-axis maturation may be a major contributor to hormonal variation at this age, and BMI exerts its influence through insulin resistance and adipokines that are more prominent at higher adiposity levels than observed here. This interpretation is consistent with Zabiegło et al. (16), who found BMI effects on AMH most pronounced above BMI 27–30 kg/m². This distinction is particularly relevant in clinical practice, as it suggests that age-related neuroendocrine maturation may outweigh adiposity-related effects in early PCOS phenotypes.

The clinical phenotype aligns with published adolescent PCOS series. Oligomenorrhoea/amenorrhoea was the dominant menstrual presentation (78.0%) and increased progressively with age (Group 1: 68.0% → Group 4: 84.1%; p=0.013), suggesting persistent menstrual irregularity rather than the transient adolescent gynaecological adjustment phase. Oligomenorrhoeic patients had higher LH/FSH ratio (2.00 vs 1.90, p=0.053) and AMH (4.70 vs 4.40 ng/mL, p=0.029) but lower total testosterone, consistent with the predominance of phenotype D (non-hyperandrogenic) within this cohort (37.6%). Hirsutism, in contrast, was strongly associated with elevated total testosterone (Mann-Whitney p<0.001), confirming ovarian androgen-driven phenotypic expression. Jakubowska-Kowal et al. reported oligomenorrhoea prevalences of 40–65% in European adolescent PCOS cohorts (10).

Dysmenorrhoea was present in 22.0% and showed no significant age gradient or hormonal association. This is clinically coherent: primary dysmenorrhoea is a prostaglandin-mediated phenomenon linked to ovulatory cycles, whereas anovulatory bleeding in PCOS produces menstrual pain through oestrogen-driven endometrial hyperproliferation and prostaglandin dysregulation rather than through the primary endocrine phenotype (27). The absence of a BMI association further supports the view that dysmenorrhoea in this cohort reflects individual pain sensitivity rather than metabolic burden.

Hirsutism (mFG ≥8) was present in 13.0% of the cohort — consistent with Turkish adolescent PCOS series using the same threshold (19, 28). Although group-level prevalence differences did not reach statistical significance (χ²=5.1, p=0.166), patient-level analyses demonstrated a strong biochemical–clinical coupling: hirsutism was significantly associated with higher total testosterone (71.8 vs 55.2 ng/dL, p<0.001) but not DHEA-S (p=0.833), confirming ovarian rather than adrenal androgen dominance. In multivariable logistic regression with EPV = 38.0, total testosterone remained the sole significant predictor after adjustment for both age and BMI (OR 1.07 per ng/dL, equivalent to an approximately two-fold increase per 10 ng/dL). The non-significance of BMI in the hirsutism model (OR 1.024, p=0.291) indicates that the testosterone–hirsutism relationship is not mediated by adiposity in this adolescent and young adult cohort. This observed association should be interpreted as reflecting the dominant measurable androgen signal within the available biochemical panel, rather than a comprehensive assessment of androgenicity — a nuance further addressed among the limitations below. The hirsutism model demonstrated good, internally validated discrimination (apparent AUROC 0.818, 95% CI 0.785–0.850; optimism-corrected 0.815), with a Brier score of 0.102. As set out in the Results, the apparent calibration slope (1.00) and intercept (0.00) are mathematical identities of the maximum-likelihood fit rather than evidence of calibration, the out-of-sample calibration slope was 0.965, and the significant Hosmer–Lemeshow test (p<0.001) reflects the near-complete separation on total testosterone, with no events in the lowest five deciles of predicted risk (**Table 7**). A parallel sensitivity analysis using biochemical hyperandrogenism (total testosterone >55 ng/dL) as the outcome confirmed that age was an inverse predictor (OR 0.896 per year, 95% CI 0.833–0.964, p=0.003) — concordant with the mid-adolescent peak — whereas BMI remained non-significant. The 100% concordance of clinical hirsutism with biochemical hyperandrogenism (all 114 hirsutism patients had total testosterone >55 ng/dL) provides convergent validation across the two operational definitions of hyperandrogenism.

The mid-adolescent enrichment across LH/FSH ratio, total testosterone, and DHEA-S was evident in the full cohort and BMI-adjusted models, although the DHEA-S peak was more sensitive to phenotype restriction than the AMH signal: BMI-adjusted multivariable median regression models showed that the Group 2 effect persisted for each primary endpoint with negligible coefficient changes after BMI adjustment, supporting an HPO-axis maturation interpretation. GnRH pulse frequency is maximal peri-menarche, and PCOS hyperandrogenism further impairs normalisation (13, 29); the bimodal age–hormone correlation pattern visualised in **Figure 1** is consistent with this neuroendocrine framework. When the cohort was re-stratified by gynaecologic age (years since menarche), effect sizes were attenuated relative to chronological-age stratification (for AMH, ϵ² 0.203→0.055), although chronological age remained the dominant predictor in joint multivariable models including chronological age, age at menarche and BMI; these cross-sectional analyses should therefore be read as consistent with, rather than demonstrating, HPO-axis maturation. The lower DHEA-S values observed in older subgroups (ρ=−0.253; **Figure 1**) are consistent with the progressive shift from peri-adrenarchal/early-adolescent adrenal androgen activity towards ovarian-dominant androgenesis in later adolescence and adulthood (30, 31).

The age-stratified hormonal profile observed in this cohort can be interpreted within the framework of the recent global consensus to rename PCOS as polyendocrine metabolic ovarian syndrome (PMOS) (5). The four primary hormonal markers analysed here map directly onto the three endocrine domains that motivated the renaming: LH/FSH ratio reflects central neuroendocrine (hypothalamic–pituitary) dysregulation; AMH and total testosterone reflect ovarian endocrine activity; and DHEA-S reflects adrenal androgen activity. The simultaneous, statistically significant variation of all four markers across age subgroups, with concurrent peaking in mid-adolescents and preservation of the AMH peak in BMI-adjusted multivariable median regression and in the strict Phenotype A subset, provides within-cohort empirical support for the polyendocrine pathophysiological framing that the renamed nomenclature is intended to capture. The fact that the largest effect size (ϵ²=0.203) was observed for AMH — a marker that is not part of the Rotterdam diagnostic criteria — further suggests that an ovary-only framing of the condition is incomplete: the most robust within-cohort signal in our data is carried by an ovarian endocrine marker that lies outside the historical Rotterdam definition, and is preserved across phenotype restriction and BMI adjustment. The differential behaviour of the four markers under phenotype restriction is also informative for the PMOS framing: while the AMH peak remained robust within Phenotype A, the DHEA-S Group 2 peak attenuated, indicating that the ovarian and adrenal endocrine domains contribute heterogeneously to the polyendocrine pattern across phenotypes — a within-PCOS/PMOS heterogeneity that an exclusively ovarian nomenclature would tend to mask. The metabolic dimension of the PMOS framing was only partially testable in this cohort: although BMI rose across age subgroups, the BMI–hormone correlations were uniformly weak and did not materially explain primary hormonal variation in adjusted analyses, and systematically available insulin, SHBG, free androgen index, and waist circumference data were not available, precluding direct interrogation of the metabolic–endocrine interplay that motivates the “metabolic” term in the renamed nomenclature. Prospective adolescent PCOS/PMOS cohorts incorporating these metabolic measurements alongside age- and gynaecologic-age-stratified hormonal profiling will be needed to fully evaluate whether the polyendocrine and metabolic dimensions of the PMOS framework behave concordantly across pubertal development, and the present findings — derived in the early months following the renaming — may inform the 2028 update of the International PCOS/PMOS Guideline with respect to age-specific hormonal interpretation in adolescents.

AMH had the largest age-related effect size among the primary hormonal endpoints (ϵ²=0.203). The P95 in Group 2 (8.25 ng/mL) exceeds that of Group 4 (5.91 ng/mL) by 39%, despite the observed BMI gradient. The 2023 ESHRE guideline’s caution against AMH as a standalone adolescent PCOS criterion (15) is supported: a fixed ≥4.5 ng/mL threshold classifies 70.0% of Group 2 as elevated but only 23.0% of Group 1. This pronounced age-dependence aligns with prior reports advocating age-specific hormonal thresholds for PCOS diagnosis in adolescents (32–35). The strong AMH–LH/FSH correlation (ρ=+0.741; per-group ρ=0.678–0.773) reflects the AMH–FSH suppression loop (36, 37) and enhanced GnRH neuronal sensitivity (38). These within-PCOS percentile distributions complement the previously published AMH reference data derived from healthy Turkish adolescent girls (39), providing clinicians with population-matched intra-PCOS benchmarks that are not directly comparable to healthy-population reference intervals. PubMed-indexed Turkish adolescent PCOS cohorts published to date have included between approximately 40 and 90 PCOS cases (e.g., Tokmak et al., 2015, n=43; Karakaş et al., 2015, n=40; Dursun & Güven 2016, n=49; Aydoğmuş et al., 2017, n=77), and the largest published Turkish PCOS cohort overall (Ateş et al., 2013, n=410) comprised adult women rather than adolescents. The present cohort (n=878 adolescent and young adult patients) therefore represents, to our knowledge, the largest single-centre age-stratified Turkish adolescent and young adult PCOS cohort published to date, and the first to provide age-stratified within-PCOS empirical centile distributions for LH/FSH ratio, AMH, and total testosterone in this population.

Rotterdam phenotype distribution varied across age subgroups: Group 2 (mid-adolescents) showed the most balanced representation across all four phenotypes, with hyperandrogenic phenotypes (A + B + C) accounting for 69.6% of this subgroup — the highest proportion across all groups. This pattern aligns with the mid-adolescent hormonal peak documented in the primary analyses and supports the concept that mid-adolescence represents the phenotypically most florid stage of PCOS expression. Mechanistically, this transient neuroendocrine state — characterised by maximal GnRH pulse activity and heightened ovarian sensitivity to gonadotropins — may constitute a diagnostic window during which biochemical and clinical features are most detectable. Recognition of this temporal window has implications for both the timing of adolescent PCOS assessment and the age-specific interpretation of biochemical thresholds, supporting the 2023 ESHRE recommendation that adult-derived cut-offs should not be uncritically extrapolated to adolescents (15).

A pre-specified sensitivity analysis restricted to the strict Phenotype A subset (full Rotterdam triad; n=207) provided further evidence that the mid-adolescent peak reflects a biologically plausible pattern rather than diagnostic-stage selection bias, while also highlighting which endpoints are most robust. The AMH peak was almost completely preserved within Phenotype A (ϵ²=0.193 versus 0.203 in the full cohort; 5% attenuation). The DHEA-S age-gradient remained statistically significant (ϵ²=0.072), but the Group 2 peak was attenuated within Phenotype A: Group 1 and Group 2 medians were closely comparable (298.5 and 291.0 µg/dL, respectively), with the gradient driven principally by the lower values in older subgroups. AMH is not part of the Rotterdam definition, so its persistence in the homogeneous Phenotype A subset constitutes the strongest within-cohort support for an HPO-axis maturation interpretation rather than a referral-pattern artefact. By contrast, ϵ² values for LH/FSH ratio (0.098 → 0.043) and total testosterone (0.064 → 0.028) were attenuated by approximately 50% within Phenotype A, although Group 2 remained the highest-ranking subgroup for both endpoints. The total testosterone attenuation is partially attributable to a definitional artefact: Phenotype A requires fulfilment of the hyperandrogenism criterion (total testosterone >55 ng/dL or modified Ferriman–Gallwey ≥8), and within the Group 1 Phenotype A subset (n=18), all 18 patients met the criterion via biochemical hyperandrogenism alone (none via clinical hirsutism), elevating the Group 1 baseline median total testosterone from 55.1 ng/dL (full cohort) to 60.2 ng/dL (Phenotype A) and thereby narrowing the Group 2–Group 1 contrast. The LH/FSH attenuation is not a definitional artefact (LH/FSH is not used in Rotterdam classification) but reflects phenotype enrichment: Phenotype A patients are by selection more uniformly hyperandrogenic across age groups, and gonadotropin-driven ovarian androgen production co-travels with this phenotypic homogeneity. Taken together, these findings support a nuanced interpretation: the mid-adolescent AMH peak is the most robust biological signal, supported across phenotype-restricted and BMI-adjusted analyses; the gonadotropin and testosterone peaks, while real in the full cohort, are partly attributable to the higher prevalence of hyperandrogenic phenotypes in Group 2 (69.6%, the highest of any subgroup); and the DHEA-S peak, while supported in BMI-adjusted regression, is more sensitive to phenotype restriction.

Oestradiol had the largest overall effect size (ϵ²=0.390, ρ=+0.569), reflecting progressive oestrogenisation superimposed on the PCOS milieu. Prolactin showed a borderline positive age association (ρ=+0.069, p=0.042, ϵ²=0.004) of uncertain clinical significance. TSH and fT4 were stable (ϵ²≤0.004), indicating thyroid monitoring need not be age-stratified.

These findings may be particularly relevant in adolescent outpatient settings where comprehensive androgen profiling is unavailable, as total testosterone alone may guide hirsutism evaluation.

This study has several methodological strengths. The large sample (n=878) constitutes one of the largest Turkish adolescent and young adult PCOS cohorts to date, with a detailed laboratory panel, concomitant BMI, and novel age-stratification. Although the absence of a healthy control group limits external comparison, this design allowed a focused within-PCOS phenotypic analysis that isolates age-related variation across adolescence. *Post-hoc* criterion-level re-abstraction confirmed Rotterdam criteria fulfilment in 94.5% of the cohort, with a balanced phenotype distribution (A: 23.6%, B: 14.8%, C: 18.6%, D: 37.6%) consistent with published PCOS series, substantially mitigating concerns about diagnostic misclassification in this retrospective design. Of particular note, standardisation of blood sampling to the early follicular phase (days 2–5) — achieved either through spontaneous menstruation or progesterone-withdrawal bleeding in anovulatory patients — ensures that FSH, LH, oestradiol, and 17-OHP values reflect true basal gonadotropin and steroid concentrations rather than cycle-phase artefacts, rarely reported in retrospective PCOS series. Future prospective studies integrating dynamic endocrine testing and comprehensive metabolic profiling are warranted to further elucidate the interplay between neuroendocrine maturation and metabolic factors in adolescent PCOS.

This study has several limitations that should be considered when interpreting the findings. The retrospective single-centre design relied on clinician-assigned PCOS diagnoses extracted from the Hospital Information System; although a pre-specified sensitivity analysis restricted to the 2023 ESHRE adolescent guideline-concordant subset (n=299; concurrent oligo-/amenorrhoea and clinical or biochemical hyperandrogenism) confirmed all principal findings, phenotype D and single-criterion patients may not fully meet the more stringent adolescent diagnostic criteria, and results in those subgroups should be interpreted as descriptive of clinician-assigned PCOS rather than guideline-confirmed adolescent PCOS. Because only 299 of 733 adolescents (40.8%) met the strict 2023 guideline-concordant adolescent criteria, the full cohort should be interpreted as a real-world clinician-assigned PCOS cohort rather than a uniformly guideline-confirmed adolescent PCOS population. The absence of a healthy adolescent control group from the same population limits direct comparison with non-PCOS populations, and we cannot determine whether the mid-adolescent peak we describe represents a PCOS-specific phenomenon or a feature of normal pubertal hormonal patterns that is amplified in PCOS. Of the 350 patients excluded from the analytic cohort (detailed in Methods and **Supplementary Figure 1**), the majority (271/350, 77.4%) were excluded for incomplete data capture (incomplete laboratory panel n=156; BMI not calculable n=87; missing age or diagnosis date n=28) rather than for clinical or biochemical phenotype features; these exclusions therefore primarily reflect operational completeness of routine adolescent gynaecology documentation rather than systematic clinical selection. Specific characteristics of excluded patients beyond exclusion category were not retrievable from the Hospital Information System within the present analytic timeframe, and we cannot fully exclude residual selection bias arising from differential completeness of the panel across phenotypes; however, the predominantly administrative nature of the exclusions suggests, but cannot prove, that major clinical selection bias was limited. Regarding anthropometric and clinical assessment, WHO adult BMI thresholds were applied to participants aged <18 years as a pragmatic simplification compatible with retrospective HIS data, although a pre-specified WHO 2007 BMI-for-age z-score sensitivity analysis confirmed robustness of all principal findings; BMI measurement protocol variability inherent to retrospective HIS records cannot be excluded. The modified Ferriman–Gallwey hirsutism score was assessed by attending clinicians under a standardised protocol, but inter-rater reliability was not formally assessed — a particular concern in adolescents in whom terminal hair development may be incomplete — and the 2-year post-menarche criterion could not be verified for eight patients aged 12–13 years. The mFG ≥8 cut-off and the biochemical hyperandrogenism threshold (total testosterone >55 ng/dL) were each recorded independently in the institutional records, but because clinicians had access to both at the time of diagnostic decision-making, the observed perfect concordance between clinical hirsutism (n=114) and biochemical hyperandrogenism in the same patients should be interpreted as convergent validity of the two operational definitions in routine practice rather than as independent corroboration. Importantly, although age at menarche and gynaecologic age were available for the full cohort and analysed in a dedicated sensitivity analysis (**Supplementary Table 5**), Tanner stage was not systematically retrievable from the Hospital Information System; chronological age therefore served as the primary stratification variable, and although the gynaecologic-age sensitivity analysis showed that hormonal patterns were also significant when stratified by time since menarche, mechanistic interpretations regarding HPO-axis maturation should be regarded as biologically plausible and supported by both age- and gynaecologic-age stratification rather than as directly measured. With respect to biochemical assessment, key parameters were absent from the available panel: SHBG and free androgen index (FAI) measurements were not systematically available, a meaningful limitation given that Özer et al. and Büyükyılmaz et al. (40, 41) recently demonstrated that FAI and SHBG outperform total testosterone in distinguishing PCOS from anovulatory cycles in Turkish adolescents; this is particularly relevant for the hirsutism logistic regression model, in which FAI may have captured additional variance beyond total testosterone, and for the interpretation of androgen bioavailability across the BMI gradient. Insulin and waist circumference data were also not systematically available, precluding comprehensive assessment of insulin resistance, metabolic phenotyping, and the interaction between adiposity and the hormonal profile that has been described in adult PCOS. BMI alone is a crude surrogate for adiposity and metabolic burden, and the observation that the BMI gradient did not materially explain the primary hormonal variation should therefore be interpreted as referring to BMI as a single anthropometric measure rather than to metabolic factors in general; insulin resistance, SHBG, FAI, waist circumference, and lipid profiles could each independently modify the hormonal patterns described here. Total testosterone was measured by automated electrochemiluminescence immunoassay (Roche Elecsys Testosterone II) rather than by liquid chromatography–tandem mass spectrometry (LC-MS/MS), the method endorsed by the 2023 ESHRE guideline as the reference standard, so absolute testosterone thresholds in adolescents should be interpreted with appropriate caution, although the Elecsys Testosterone II assay has demonstrated acceptable correlation with LC-MS/MS in published method-comparison studies. Free testosterone was measured by direct automated immunoassay rather than calculated from total testosterone and SHBG (which was not available) or measured by equilibrium dialysis, and the 2023 ESHRE guideline cautions against direct free testosterone immunoassays owing to known performance limitations; free testosterone results from this cohort should accordingly be considered exploratory rather than diagnostically definitive. Finally, the use of baseline 17-OHP thresholds (>2.5 ng/mL, or 2.0–2.5 ng/mL with additional clinical features) for exclusion of non-classic congenital adrenal hyperplasia, rather than ACTH stimulation testing, may have introduced minor classification uncertainty for patients in the 2.0–2.5 ng/mL grey zone.

Several additional considerations relate to treatment exposure and unmeasured confounders. Detailed pre-sampling pharmacological treatment history (combined oral contraceptives, anti-androgens, metformin, or insulin-sensitising agents) was not systematically extractable from HIS records, although the index-visit sampling design — capturing patients at the point of initial PCOS diagnosis — likely minimised exposure to prior PCOS-directed pharmacotherapy in this adolescent outpatient population. The progesterone-withdrawal protocol used for anovulatory patients, while ensuring early-follicular-phase sampling conditions, introduces a minor pharmacological stimulus that may theoretically influence the hormonal milieu in a small subset. Residual confounding from unmeasured variables — including lifestyle, dietary patterns, exercise, sleep, family history, and ethnicity beyond Turkish identification — cannot be fully excluded.

## Conclusions

In 878 adolescent and young adult patients with clinician-assigned PCOS (aged 10–26 yr), BMI rose across age subgroups but did not materially explain the primary hormonal variation in adjusted models, consistent with HPO maturation as a possible major contributing mechanism. The higher mid-adolescent AMH values (the largest effect size, ϵ²=0.203) were preserved within the strict Phenotype A subset (full Rotterdam triad; n=207), within the non-hyperandrogenic Phenotype D subset (ϵ²=0.197), and after BMI-adjusted multivariable median regression, supporting it as the most robust hormonal signal in this cohort; the DHEA-S age-gradient was preserved in BMI-adjusted analyses but the Group 2 peak attenuated within the strict Phenotype A subset, suggesting that the AMH signal is more robust to phenotype enrichment than the adrenal-androgen signal. Total testosterone was the strongest hormonal correlate of hirsutism within the available biochemical panel, after adjustment for age and BMI, with the logistic regression model showing good, internally validated discrimination (optimism-corrected AUROC 0.815); this finding supports its use as a clinically relevant biochemical correlate, pending confirmation in prospective studies and in panels that include SHBG and free androgen index. Oligomenorrhoea/amenorrhoea (78.0%) was the dominant menstrual abnormality and was accompanied by higher LH/FSH ratio and AMH, whereas hirsutism was most closely associated with total testosterone. The empirical P5–P95 centile distributions constitute, to our knowledge based on a targeted PubMed search, the largest Turkish age-stratified within-PCOS intra-cohort centile dataset for LH/FSH ratio, AMH, and total testosterone to date, substantially exceeding the 40–90-patient adolescent PCOS cohorts previously reported in the Turkish literature. These findings, derived from a cross-sectional cohort without healthy controls, highlight the importance of age-specific interpretation of hormonal profiles in adolescent and young adult patients evaluated for PCOS — the condition for which a multisystem renaming to polyendocrine metabolic ovarian syndrome has recently been proposed — and should be regarded as hypothesis-generating with respect to underlying pubertal mechanisms.

## Data Availability

The datasets presented in this article are not readily available because the dataset contains sensitive adolescent health data extracted from the Hospital Information System and is subject to patient confidentiality and institutional privacy restrictions. Public sharing of the raw dataset is not permitted under the conditions of ethics committee approval (Clinical Research Ethics Committee of Ankara Etlik City Hospital; approval reference: AEŞH-BADEK1-2026-425; approval date: 6 May 2026), which authorised the use of these data for the present retrospective analysis only, with full anonymisation. De-identified aggregate data or a limited de-identified dataset supporting the conclusions may be made available from the corresponding author upon reasonable request from qualified researchers, subject to institutional and ethics committee approval and a data-sharing agreement. Requests to access the datasets should be directed to Sait Erbey, saiterbey@gmail.com.

## References

[1] March WA Moore VM Willson KJ Phillips DI Norman RJ Davies MJ . The prevalence of polycystic ovary syndrome in a community sample assessed under contrasting diagnostic criteria. Hum Reprod. (2010) 25:544–51. doi: 10.1093/humrep/dep399 19910321

[2] Bozdag G Mumusoglu S Zengin D Karabulut E Yildiz BO . The prevalence and phenotypic features of polycystic ovary syndrome: a systematic review and meta-analysis. Hum Reprod. (2016) 31:2841–55. doi: 10.1093/humrep/dew218 27664216

[3] Amiri M Hatoum S Buyalos RP Sheidaei A Azziz R . The influence of study quality, age, and geographic factors on PCOS prevalence: a systematic review and meta-analysis. J Clin Endocrinol Metab. (2025) 110:2082–103. doi: 10.1210/clinem/dgae917 39758024

[4] Neven ACH Forslund M Ranashinha S Mousa A Tay CT Peña A . Prevalence and accurate diagnosis of polycystic ovary syndrome in adolescents across world regions: a systematic review and meta-analysis. Eur J Endocrinol. (2024) 191:S15–27. doi: 10.1093/ejendo/lvae125 39353075

[5] Teede HJ Bahri Khomami M Morman R Laven JSE Joham AE Costello MF . Polyendocrine metabolic ovarian syndrome, the new name for polycystic ovary syndrome: a multistep global consensus process. Lancet. (2026) 407:2329–39. doi: 10.1016/S0140-6736(26)00717-8 42119588

[6] Azziz R Carmina E Chen Z Dunaif A Laven JS Legro RS . Polycystic ovary syndrome. Nat Rev Dis Primers. (2016) 2:16057. doi: 10.1038/nrdp.2016.57 27510637

[7] Amisi CA . Markers of insulin resistance in polycystic ovary syndrome women: an update. World J Diabetes. (2022) 13:129–49. doi: 10.4239/wjd.v13.i3.129 35432749 PMC8984569

[8] Lim SS Davies MJ Norman RJ Moran LJ . Overweight, obesity and central obesity in women with polycystic ovary syndrome: a systematic review and meta-analysis. Hum Reprod Update. (2012) 18:618–37. doi: 10.1093/humupd/dms030 22767467

[9] McCartney CR Blank SK Prendergast KA Chhabra S Eagleson CA Helm KD . Obesity and sex steroid changes across puberty: evidence for marked hyperandrogenemia in pre- and early pubertal obese girls. J Clin Endocrinol Metab. (2007) 92:430–6. doi: 10.1210/jc.2006-2002 17118995 PMC2196134

[10] Jakubowska-Kowal K Skrzyńska K Gawlik-Starzyk A . Treatment and complications of PCOS in adolescents — what's new in 2023? Front Endocrinol (Lausanne). (2024) 15:1436952. doi: 10.3389/fendo.2024.1436952 39415788 PMC11479989

[11] Witchel SF Oberfield SE Peña AS . Polycystic ovary syndrome: pathophysiology, presentation, and treatment with emphasis on adolescent girls. J Endocr Soc. (2019) 3:1545–73. doi: 10.1210/js.2019-00078 31384717 PMC6676075

[12] Assens M Dyre L Henriksen LS Brocks V Sundberg K Jensen LN . Menstrual pattern, reproductive hormones, and transabdominal 3D ultrasound in 317 adolescent girls. J Clin Endocrinol Metab. (2020) 105:e3257–66. doi: 10.1210/clinem/dgaa355 32506132

[13] Blank SK McCartney CR Marshall JC . The origins and sequelae of abnormal neuroendocrine function in polycystic ovary syndrome. Hum Reprod Update. (2006) 12:351–61. doi: 10.1093/humupd/dml017 16670102

[14] Rosenfield RL Ehrmann DA . The pathogenesis of polycystic ovary syndrome (PCOS): the hypothesis of PCOS as functional ovarian hyperandrogenism revisited. Endocr Rev. (2016) 37:467–520. doi: 10.1210/er.2015-1104 27459230 PMC5045492

[15] Teede HJ Tay CT Laven JJE Dokras A Moran LJ Piltonen TT . Recommendations from the 2023 international evidence-based guideline for the assessment and management of polycystic ovary syndrome. J Clin Endocrinol Metab. (2023) 108:2447–69. doi: 10.1210/clinem/dgad463 37580314 PMC10505534

[16] Zabiegło E Jach R Piróg M . Age and BMI-related changes in hormonal profile in women with polycystic ovary syndrome (PCOS): Association with infertility. Int J Gynaecol Obstet. (2025) 171:353–61. doi: 10.1002/ijgo.70160 40257455

[17] von Elm E Altman DG Egger M Pocock SJ Gøtzsche PC Vandenbroucke JP . The Strengthening the Reporting of Observational Studies in Epidemiology (STROBE) statement: guidelines for reporting observational studies. J Clin Epidemiol. (2008) 61:344–9. doi: 10.1016/j.jclinepi.2007.11.008 18313558

[18] Sawyer SM Azzopardi PS Wickremarathne D Patton GC . The age of adolescence. Lancet Child Adolesc Health. (2018) 2:223–8. doi: 10.1016/S2352-4642(18)30022-1 30169257

[19] Yildiz BO Bolour S Woods K Moore A Azziz R . Visually scoring hirsutism. Hum Reprod Update. (2010) 16:51–64. doi: 10.1093/humupd/dmp024 19567450 PMC2792145

[20] Bizuneh AD Joham AE Teede H Mousa A Earnest A Hawley JM . Evaluating the diagnostic accuracy of androgen measurement in polycystic ovary syndrome: a systematic review and diagnostic meta-analysis to inform evidence-based guidelines. Hum Reprod Update. (2025) 31:48–63. doi: 10.1093/humupd/dmae028 39305127 PMC11696697

[21] Elhassan YS Hawley JM Cussen L Abbara A Clarke SA Kempegowda P . Society for Endocrinology clinical practice guideline for the evaluation of androgen excess in women. Clin Endocrinol (Oxf). (2025) 103:540–66. doi: 10.1111/cen.15265 40364581 PMC12413683

[22] Lakens D . Calculating and reporting effect sizes to facilitate cumulative science: a practical primer for t-tests and ANOVAs. Front Psychol. (2013) 4:863. doi: 10.3389/fpsyg.2013.00863 24324449 PMC3840331

[23] Jakubowska-Kowal KM Skrzynska KJ Gawlik-Starzyk AM . Prevalence and diagnosis of polycystic ovary syndrome (PCOS) in adolescents — what's new in 2023? Systematic review. Ginekol Pol. (2024) 95:643–9. doi: 10.5603/gpl.98849 39140356

[24] Franks S . Polycystic ovary syndrome in adolescents. Int J Obes (Lond). (2008) 32:1035–41. doi: 10.1038/ijo.2008.61 18458678

[25] Zhang Y Yang K Fan T Zheng D Liu H . Diagnosis and treatment of adolescent polycystic ovary syndrome: a review. Int J Womens Health. (2025) 17:459–74. doi: 10.2147/IJWH.S506498 39995885 PMC11847718

[26] Dewailly D Andersen CY Balen A Broekmans F Dilaver N Fanchin R . The physiology and clinical utility of anti-Mullerian hormone in women. Hum Reprod Update. (2014) 20:370–85. doi: 10.1093/humupd/dmt062 24430863

[27] Dawood MY . Primary dysmenorrhea: advances in pathogenesis and management. Obstet Gynecol. (2006) 108:428–41. doi: 10.1097/01.AOG.0000230214.26638.0c 16880317

[28] Azziz R Carmina E Sawaya ME . Idiopathic hirsutism. Endocr Rev. (2000) 21:347–62. doi: 10.1210/edrv.21.4.0401 10950156

[29] Eagleson CA Gingrich MB Pastor CL Arora TK Burt CM Evans WS . Polycystic ovarian syndrome: evidence that flutamide restores sensitivity of the gonadotropin-releasing hormone pulse generator to inhibition by estradiol and progesterone. J Clin Endocrinol Metab. (2000) 85:4047–52. doi: 10.1210/jcem.85.11.6992 11095431

[30] Rainey WE Nakamura Y . Regulation of the adrenal androgen biosynthesis. J Steroid Biochem Mol Biol. (2008) 108:281–6. doi: 10.1016/j.jsbmb.2007.09.015 17945481 PMC2699571

[31] Azziz R Black V Hines GA Fox LM Boots LR . Adrenal androgen excess in the polycystic ovary syndrome: sensitivity and responsivity of the hypothalamic-pituitary-adrenal axis. J Clin Endocrinol Metab. (1998) 83:2317–23. doi: 10.1210/jcem.83.7.4948 9661602

[32] Khashchenko E Uvarova E Vysokikh M Ivanets T Krechetova L Tarasova N . The relevant hormonal levels and diagnostic features of polycystic ovary syndrome in adolescents. J Clin Med. (2020) 9:1831. doi: 10.3390/jcm9061831 32545404 PMC7355484

[33] Timur HT Cimrin D Gursoy Doruk O Dogan OE . Determining the age group-based cut-off values of serum anti-Müllerian hormone concentrations to diagnose polycystic ovary syndrome. Curr Med Res Opin. (2023) 39:855–63. doi: 10.1080/03007995.2023.2204768 37074782

[34] Ramezani Tehrani F Rahmati M Mahboobifard F Firouzi F Hashemi N Azizi F . Age-specific cut-off levels of anti-Müllerian hormone can be used as diagnostic markers for polycystic ovary syndrome. Reprod Biol Endocrinol. (2021) 19:76. doi: 10.1186/s12958-021-00755-8 34022904 PMC8140506

[35] van der Ham K Laven JSE Tay CT Mousa A Teede H Louwers YV . Anti-Müllerian hormone as a diagnostic biomarker for polycystic ovary syndrome and polycystic ovarian morphology: a systematic review and meta-analysis. Fertil Steril. (2024) 122:727–39. doi: 10.1016/j.fertnstert.2024.05.163 38944177

[36] Pellatt L Rice S Mason HD . Anti-Müllerian hormone and polycystic ovary syndrome: a mountain too high? Reproduction. (2010) 139:825–33. doi: 10.1530/REP-09-0415 20207725

[37] Broer SL Broekmans FJ Laven JS Fauser BC . Anti-Müllerian hormone: ovarian reserve testing and its potential clinical implications. Hum Reprod Update. (2014) 20:688–701. doi: 10.1093/humupd/dmu020 24821925

[38] Tata B Mimouni NEH Barbotin AL Malone SA Loyens A Pigny P . Elevated prenatal anti-Müllerian hormone reprograms the fetus and induces polycystic ovary syndrome in adulthood. Nat Med. (2018) 24:834–46. doi: 10.1038/s41591-018-0035-5 29760445 PMC6098696

[39] Özçivit Erkan İB Öncül M Başıbüyük Z Çebi C Çepni İ . Serum anti-Mullerian hormone levels in Turkish girls aged 18 and younger for ovarian reserve determination. J Turk Ger Gynecol Assoc. (2024) 25:138–43. doi: 10.4274/jtgga.galenos.2024.2023-8-5 38775713 PMC11576653

[40] Özer E Taş D Çakır Gündoğan S . Clinical utility of FAI and SHBG in differentiating PCOS from anovulatory cycles in adolescent girls. Front Pediatr. (2025) 13:1581060. doi: 10.3389/fped.2025.1581060 40861046 PMC12370747

[41] Büyükyılmaz G Koca SB Toksoy Adıgüzel K Boyraz M Gurbuz F . The role of the AMH, SHBG, free androgen index and LH/FSH ratio in the diagnosis of polycystic ovary syndrome in adolescents. Turk J Pediatr Dis. (2024) 18:34–40. doi: 10.12956/tchd.1347807

