# Supplementary material for: Age-stratified hormonal profiles in adolescent and young adult PCOS/PMOS: higher mid-adolescent AMH largely unexplained by BMI
Source: Front Endocrinol (Lausanne). 2026 Aug 5;17:1890066. doi: 10.3389/fendo.2026.1890066 (PMC13485579; doi:10.3389/fendo.2026.1890066)
Supplement: Supplementary file 1 [file DataSheet1.docx]

**Supplementary Material**

*for the manuscript:*

*Age-Stratified Hormonal Profiles in Adolescent and Young Adult PCOS/PMOS: Higher Mid-Adolescent AMH Largely Unexplained by BMI*

**Contents**

- **Supplementary Figure 1.** STROBE flow diagram of participant selection (this file, below).
- **Supplementary Tables 1–5.** Sensitivity analyses — 1, WHO 2007 BMI-for-age reclassification; 2, BMI-adjusted multivariable median (quantile) regression; 3, strict Phenotype A subset; 4, median-split temporal analysis; 5, gynaecologic-age (post-menarche) analysis.
- **Supplementary File 1.** STROBE Statement — checklist of items for reports of cohort studies.
- **Supplementary File 2.** RECORD Statement — extension of STROBE for routinely-collected health data.

*All supplementary items for this manuscript are provided together in this single Supplementary Material file. Each item retains the label used in the main manuscript text.*

**Supplementary Figure 1**

*Supplementary Figure 1 | STROBE flow diagram of participant selection. From 11,324 consecutive adolescent outpatient visits, 1,228 patients received a clinician-assigned PCOS diagnosis; 350 were excluded (ten pre-specified categories) to yield the final analytic cohort of 878 patients.*

**Supplementary Tables**

*Age-Stratified Hormonal Profiles in Adolescent and Young Adult PCOS/PMOS: Higher Mid-Adolescent AMH Largely Unexplained by BMI*

**Supplementary Table 1. WHO 2007 BMI-for-age reclassification sensitivity analysis for participants aged <18 years.**

| **Age group** | **Threshold** | **Underweight/Thinness** | **Normal** | **Overweight** | **Obese** | **OW+Obese** |
| --- | --- | --- | --- | --- | --- | --- |
| Group 1 (≤15, n=100) | Adult WHO | 8 (8.0%) | 57 (57.0%) | 30 (30.0%) | 5 (5.0%) | 35 (35.0%) |
| Group 1 (≤15, n=100) | WHO 2007 z-score | 5 (5.0%) | 33 (33.0%) | 36 (36.0%) | 26 (26.0%) | 62 (62.0%) |
| Group 2 (16–17, n=217) | Adult WHO | 19 (8.8%) | 81 (37.3%) | 77 (35.5%) | 40 (18.4%) | 117 (53.9%) |
| Group 2 (16–17, n=217) | WHO 2007 z-score | 13 (6.0%) | 70 (32.3%) | 62 (28.6%) | 72 (33.2%) | 134 (61.8%) |

*Adult WHO thresholds: underweight <18.5 kg/m², normal 18.5–24.9, overweight 25.0–29.9, obese ≥30.0. WHO 2007 z-score thresholds: thinness z<−2 SD, normal −2 ≤ z < +1 SD, overweight +1 ≤ z < +2 SD, obese z ≥ +2 SD. BMI-for-age z-scores were calculated using published LMS parameters for girls aged 10–19 years, assigning mid-year age-in-months values to each participant.*

**Supplementary Table 2. BMI-adjusted multivariable median (quantile) regression models for primary hormonal endpoints (n=878).**

| **Endpoint** | **Predictor** | **β** | **95% CI** | **p** |
| --- | --- | --- | --- | --- |
| *LH/FSH ratio* | Intercept | +1.900 | [+1.562, +2.238] | <0.001 |
|  | Group 2 vs 1 (ref) | +0.400 | [+0.191, +0.609] | <0.001 |
|  | Group 3 vs 1 (ref) | +0.100 | [−0.093, +0.293] | 0.310 |
|  | Group 4 vs 1 (ref) | −0.300 | [−0.527, −0.073] | 0.010 |
|  | BMI (per kg/m²) | −0.0001 | [−0.012, +0.012] | 1.000 |
| *AMH (ng/mL)* | Intercept | +3.464 | [+2.722, +4.207] | <0.001 |
|  | Group 2 vs 1 (ref) | +1.721 | [+1.261, +2.181] | <0.001 |
|  | Group 3 vs 1 (ref) | +1.408 | [+0.983, +1.833] | <0.001 |
|  | Group 4 vs 1 (ref) | −0.209 | [−0.709, +0.291] | 0.413 |
|  | BMI (per kg/m²) | +0.006 | [−0.021, +0.032] | 0.679 |
| *Total testosterone (ng/dL)* | Intercept | +45.568 | [+36.462, +54.675] | <0.001 |
|  | Group 2 vs 1 (ref) | +8.241 | [+2.602, +13.880] | 0.004 |
|  | Group 3 vs 1 (ref) | +2.553 | [−2.659, +7.764] | 0.337 |
|  | Group 4 vs 1 (ref) | −8.033 | [−14.163, −1.903] | 0.010 |
|  | BMI (per kg/m²) | +0.427 | [+0.099, +0.755] | 0.011 |
| *DHEA-S (µg/dL)* | Intercept | +299.892 | [+266.483, +333.301] | <0.001 |
|  | Group 2 vs 1 (ref) | +20.807 | [+0.120, +41.493] | 0.049 |
|  | Group 3 vs 1 (ref) | −12.797 | [−31.916, +6.323] | 0.189 |
|  | Group 4 vs 1 (ref) | −38.098 | [−60.587, −15.609] | 0.001 |
|  | BMI (per kg/m²) | −0.397 | [−1.600, +0.806] | 0.518 |

*Median (quantile) regression models with each primary hormonal endpoint as the dependent variable, age subgroup (Group 1 as reference) as the main categorical predictor, and BMI as a continuous covariate. Group 2 (mid-adolescent) effects vs Group 1 (early adolescent) reference remained statistically significant for all four endpoints after BMI adjustment, with negligible coefficient changes (range −13.2% to +3.6%) compared with unadjusted models. Parallel log-linear regression on log-transformed hormonal values yielded concordant results, with Group 2 ratios of 1.26 (LH/FSH), 1.61 (AMH), 1.24 (total testosterone), and 1.08 (DHEA-S) versus Group 1 (all p<0.05); BMI was non-significant for LH/FSH, AMH, and DHEA-S in log-linear models (all p>0.4).*

**Supplementary Table 3. Strict Phenotype A subset sensitivity analysis: primary hormonal endpoints by age subgroup (n=207).**

| **Parameter** | **Group 1 (≤15 yr) n=18** | **Group 2 (16–17 yr) n=52** | **Group 3 (18–19 yr) n=104** | **Group 4 (≥20 yr) n=33** | **KW p / ε² (full → Phen A)** |
| --- | --- | --- | --- | --- | --- |
| LH/FSH ratio | 2.05 | 2.25 | 1.95 | 1.60 | p=0.009; ε²=0.043 (0.098 → 0.043) |
| AMH (ng/mL) | 3.95 | 5.70 | 5.05 | 3.40 | p<0.001; ε²=0.193 (0.203 → 0.193) |
| Total testosterone (ng/dL) | 60.2 | 64.2 | 62.8 | 61.1 | p=0.033; ε²=0.028 (0.064 → 0.028) |
| DHEA-S (µg/dL) | 298.5 | 291.0 | 264.5 | 241.0 | p<0.001; ε²=0.072 (0.094 → 0.072) |

*Values are within-group medians for the strict Phenotype A subset (full Rotterdam triad: oligo-/amenorrhoea + clinical or biochemical hyperandrogenism + polycystic ovarian morphology; n=207). KW, Kruskal–Wallis; ε², epsilon-squared effect size. The AMH peak was preserved with minimal attenuation, supporting its interpretation as a robust biological signal of HPO-axis maturation independent of phenotype enrichment. The DHEA-S age-gradient remained statistically significant overall, but the Group 2 peak observed in the full cohort was attenuated within Phenotype A: Group 1 and Group 2 medians (298.5 and 291.0 µg/dL, respectively) were closely comparable, with the gradient driven principally by lower values in older subgroups. The LH/FSH and total testosterone peaks were attenuated by approximately 50% within Phenotype A but Group 2 remained the highest-ranking subgroup for both endpoints; the total testosterone attenuation is partially attributable to a definitional artefact (Group 1 Phenotype A median total testosterone is elevated relative to the full cohort because all 18 G1 patients met the C2 hyperandrogenism criterion via biochemical hyperandrogenism, total testosterone >55 ng/dL, raising the Group 1 baseline median from 55.1 to 60.2 ng/dL and narrowing the Group 2–Group 1 contrast). Group 2 retained the highest median value for AMH, LH/FSH ratio, and total testosterone; for DHEA-S, the Group 2 median was marginally lower than that of Group 1 within this subset.*

**Supplementary Table 4. Median-split temporal sensitivity analysis: comparison of primary hormonal endpoints between early and late study halves.**

| **Parameter** | **Early period (n=439)** | **Late period (n=438)** | **Early vs Late MW p** | **Early-half mid-adolescent peak (ε²)** | **Late-half mid-adolescent peak (ε²)** |
| --- | --- | --- | --- | --- | --- |
| LH/FSH ratio (median) | 1.90 | 2.00 | 0.23 | 0.119 (G2 peak) | 0.067 (G2 peak) |
| AMH (ng/mL, median) | 4.60 | 4.80 | 0.11 | 0.217 (G2 peak) | 0.180 (G2 peak) |
| Total testosterone (ng/dL, median) | 58.3 | 58.4 | 0.66 | 0.076 (G2 peak) | 0.050 (G2 peak) |
| DHEA-S (µg/dL, median) | 278.0 | 287.5 | 0.02 | 0.115 (G2 peak) | 0.067 (G1 peak†) |
| BMI (kg/m², median) | 25.9 | 25.3 | 0.16 | — | — |
| Hirsutism prevalence | 11.2% | 14.8% | 0.13 | — | — |
| Oligomenorrhoea/amenorrhoea prevalence | 80.0% | 76.0% | 0.19 | — | — |

*Median split based on visit date (early period: visits ≤2025-01-24, n=439; late period: visits >2025-01-24, n=438; one patient with missing visit date excluded). MW, Mann–Whitney U test (early vs late). ε², epsilon-squared effect size for the Kruskal–Wallis test of the four-group age-subgroup comparison within each half. † In the late half, DHEA-S median is essentially identical between Group 1 (302.0) and Group 2 (299.5; 0.8% difference of negligible clinical significance); the Group 1 vs Group 2 contrast is not statistically significant by Bonferroni-corrected pairwise comparison. All four primary hormonal endpoints retained Group 2 as the highest-ranking subgroup in the early half; in the late half, Group 2 retained the peak for LH/FSH, AMH, and total testosterone, with the marginal DHEA-S exception noted above. The mid-adolescent peak was therefore preserved across both halves of the study period for the principal endpoints, indicating no meaningful temporal heterogeneity.*

**Supplementary Table 5. Gynaecologic-age (post-menarche) sensitivity analysis: primary hormonal endpoints by years since menarche.**

| **Endpoint** | **GA1: <2 yr (n=47)** | **GA2: 2–3 yr (n=136)** | **GA3: 4–6 yr (n=384)** | **GA4: ≥7 yr (n=311)** | **KW p; ε²** |
| --- | --- | --- | --- | --- | --- |
| **LH/FSH ratio** | 2.00 | 2.20 | 2.10 | 1.80 | <0.001; ε²=0.044 |
| **AMH (ng/mL)** | 4.10 | 4.60 | 5.00 | 4.30 | <0.001; ε²=0.055 |
| **Total testosterone (ng/dL)** | 55.0 | 62.2 | 59.5 | 56.2 | 0.007; ε²=0.011 |
| **DHEA-S (µg/dL)** | 289.0 | 284.5 | 293.5 | 269.0 | <0.001; ε²=0.027 |

*Values are within-stratum medians for the primary hormonal endpoints in 878 patients re-stratified by gynaecologic age (chronological age minus age at menarche). KW, Kruskal–Wallis; ε², epsilon-squared effect size for the four-stratum gynaecologic-age comparison. Effect sizes are attenuated relative to chronological-age stratification (e.g., AMH ε² 0.203 → 0.055), and Spearman correlations of the four primary hormones with gynaecologic age were weaker than with chronological age (LH/FSH ρ=−0.192 vs −0.224; AMH ρ=−0.119 vs −0.154; total testosterone ρ=−0.098 vs −0.147; DHEA-S ρ=−0.188 vs −0.253). In multivariable median (quantile) regression including chronological age, age at menarche, and BMI as joint predictors, chronological age was an independent significant predictor for all four endpoints (all p<0.05) whereas age at menarche was non-significant in every model (all p>0.25), indicating that chronological age captures the dominant signal in this cohort. The 47 patients within 2 years of menarche showed lower median values for all four endpoints than the 2–3 yr stratum, supporting the 2023 ESHRE caution that diagnostic markers may be misleading in the immediate post-menarcheal interval. Overall, hormonal patterns observed across chronological age subgroups also map qualitatively to time since menarche, providing additional support for an HPO-axis-maturation interpretation while indicating that chronological age is the stronger stratifier in this cross-sectional cohort.*

**Supplementary File 1. STROBE Checklist**

**STROBE Statement — Checklist of Items That Should Be Included in Reports of Cohort Studies**

*Manuscript: "Age-Stratified Hormonal Profiles in Adolescent and Young Adult PCOS/PMOS: Higher Mid-Adolescent AMH Largely Unexplained by BMI"*

The Strengthening the Reporting of Observational Studies in Epidemiology (STROBE) checklist below summarises how each of the 22 recommended reporting items is addressed in the present manuscript. Section references correspond to the main manuscript text.

| **Item No.** | **STROBE Recommendation** | **Description of how addressed in this manuscript** | **Section / Page** |
| --- | --- | --- | --- |
| **Title and Abstract** | | | |
| 1 (a) | Indicate the study's design with a commonly used term in the title or the abstract | The abstract identifies the design in its opening sentences as a single-centre retrospective cohort of clinician-assigned PCOS/PMOS. The title indicates an age-stratified analysis of a PCOS/PMOS cohort. | Title;  Abstract (Methods) |
| 1 (b) | Provide in the abstract an informative and balanced summary of what was done and what was found | The abstract is a single unstructured paragraph that follows the IMRaD sequence (background, cohort and methods, results, conclusions). Sample size, analytic cohort selection, statistical methods (Kruskal–Wallis, Spearman, logistic regression with EPV), key effect sizes (ε²), and the principal findings (the higher mid-adolescent AMH values; total testosterone as the only significant correlate of hirsutism) are all summarised. | Abstract |
| **Introduction** | | | |
| 2 | Background/rationale: Explain the scientific background and rationale for the investigation being reported | The Introduction establishes PCOS prevalence (6–21%), its metabolic/cardiovascular sequelae, the central role of BMI as a phenotypic modulator, and the confounding problem of co-directional age–BMI trajectories during puberty. The rationale for age-stratification in a Turkish population is explicitly stated. | Introduction, ¶1–4 |
| 3 | Objectives: State specific objectives, including any prespecified hypotheses | Objectives are stated in the Abstract — to describe how hormonal, anthropometric and clinical profiles differ across age and whether these differences are explained by the parallel BMI gradient — and expanded in the final paragraph of the Introduction. The HPO-maturation vs BMI-confounding hypothesis is pre-specified. | Abstract; Introduction, final ¶ |
| **Methods** | | | |
| 4 | Study design: Present key elements of study design early in the paper | The first paragraph of Materials and Methods states: "This was a single-centre retrospective cohort study at the Adolescent Outpatient Clinic of Ankara Etlik City Hospital." | Materials and Methods, ¶1 |
| 5 | Setting: Describe the setting, locations, and relevant dates, including periods of recruitment, exposure, follow-up, and data collection | Setting (Adolescent Outpatient Clinic, Ankara Etlik City Hospital), country (Türkiye), and full recruitment period (January 2024 – February 2026) are stated. Data were extracted from the institutional HIS within ±90 days of the diagnostic visit. | Abstract (Methods); Materials and Methods, ¶1–2 |
| 6 (a) | Participants: Give the eligibility criteria, and the sources and methods of selection of participants. Describe methods of follow-up | Source population (11,324 consecutive adolescent outpatient visits), eligibility (age 10–26 years, clinician-assigned PCOS, complete hormonal panel, calculable BMI), and complete exclusion hierarchy with counts (n=350 excluded across 10 predefined criteria) are reported. No longitudinal follow-up (cross-sectional analysis at index visit). | Materials and Methods, ¶2; Supplementary Figure 1 |
| 6 (b) | For matched studies, give matching criteria and number of exposed and unexposed | Not applicable. This is an unmatched single-cohort study without exposed/unexposed stratification; age-subgroup allocation was predefined rather than matched. | N/A |
| 7 | Variables: Clearly define all outcomes, exposures, predictors, potential confounders, and effect modifiers. Give diagnostic criteria, if applicable | Primary outcomes (LH/FSH ratio, AMH, total testosterone, DHEA-S) and secondary outcomes (BMI distribution, clinical diagnoses, correlations, hirsutism predictors, centile distributions) are defined. PCOS diagnostic criteria (modified Rotterdam and 2023 ESHRE) are specified, with explicit post-hoc criterion-level Rotterdam verification described. mFG ≥8 cut-off for hirsutism, BMI thresholds (WHO adult and WHO 2007 z-score for sensitivity), and the 17-OHP exclusion thresholds for non-classic CAH (>2.5 ng/mL, or 2.0–2.5 ng/mL with additional clinical features) are all defined. | Materials and Methods, ¶3–7 |
| 8* | Data sources/measurement: For each variable of interest, give sources of data and details of methods of assessment (measurement). Describe comparability of assessment methods if there is more than one group | Blood sampling standardised to cycle days 2–5 (early follicular phase); progesterone-withdrawal protocol for anovulatory patients specified (dydrogesterone or medroxyprogesterone acetate). All institutional hormone assays (AMH, total and free testosterone, DHEA-S, 17-OHP, FSH, LH, oestradiol, prolactin, TSH, fT4) performed on a Roche Cobas e-series automated analyser using ECLIA (Elecsys reagents; Roche Diagnostics, Mannheim, Germany) in an ISO 15189-accredited laboratory; AMH measured with the Elecsys AMH assay and total testosterone with the Elecsys Testosterone II assay. Free testosterone measured by direct automated immunoassay rather than calculated (SHBG not available); BMI measured with calibrated clinic equipment under standardised conditions. mFG assessed by attending clinician under a standardised protocol. PCOM determined by transabdominal or transvaginal ultrasound. | Materials and Methods, ¶6–8 |
| 9 | Bias: Describe any efforts to address potential sources of bias | Multiple bias-mitigation strategies are reported: (1) uniform early-follicular-phase sampling minimises cycle-phase artefacts; (2) ten exclusion criteria remove confounding endocrine comorbidities (thyroid disease, hyperprolactinaemia, non-classic CAH, POI, corticosteroids); (3) ±90-day record-linkage window limits temporal misclassification; (4) post-hoc Rotterdam criterion-level re-abstraction validates diagnostic accuracy (94.5% fulfilment); (5) WHO 2007 z-score sensitivity analysis addresses age-specific BMI classification bias; (6) sensitivity analysis excluding Group 1 addresses adolescent diagnostic uncertainty. | Materials and Methods, ¶2, 5, 9; Results (Rotterdam verification; WHO sensitivity; Group-1 sensitivity) |
| 10 | Study size: Explain how the study size was arrived at | Sample size was determined by full enumeration of all eligible patients meeting the pre-specified inclusion criteria during the defined study period (n=878 of 1,228 PCOS-diagnosed patients from 11,324 consecutive visits). No a priori power calculation was performed; however, events-per-variable for the primary logistic regression (EPV=38.0) substantially exceeds the conventional EPV ≥10 threshold. | Materials and Methods, ¶2, 9; Results ¶1 |
| 11 | Quantitative variables: Explain how quantitative variables were handled in the analyses. If applicable, describe which groupings were chosen and why | Continuous variables are reported as median [Q1–Q3] (non-parametric, given non-normal distributions). BMI was analysed both as a continuous variable (primary analyses) and categorised by WHO adult thresholds for descriptive purposes. Age was stratified into four pre-specified subgroups (≤15, 16–17, 18–19, ≥20 years) based on adolescence stage nomenclature [refs 15, 18]. Rationale for groupings is provided. | Materials and Methods, ¶4–5, 9 |
| 12 (a) | Statistical methods: Describe all statistical methods, including those used to control for confounding | Kruskal–Wallis H tests (with Bonferroni-corrected pairwise Mann–Whitney U; k=6, α=0.0083); ε² effect sizes computed as (H−k+1)/(n−k); Pearson's chi-square for categorical comparisons; Spearman's ρ for monotonic associations; multivariable logistic regression with age, total testosterone, and BMI as continuous covariates (for hirsutism, adjusting for age and BMI). Variance inflation factors (VIF) reported to exclude multicollinearity (all <2). Logistic regression performance evaluated by AUROC (with bootstrap 95% CI), Hosmer–Lemeshow goodness-of-fit, calibration slope and intercept, and Brier score. Primary analyses performed in SPSS 27.0; supplementary BMI-adjusted multivariable median (quantile) and log-linear regression analyses performed in Python 3.11 (statsmodels v0.14). | Materials and Methods, ¶9 |
| 12 (b) | Describe any methods used to examine subgroups and interactions | Four pre-specified age subgroups were analysed with Kruskal–Wallis followed by Bonferroni-corrected pairwise Mann–Whitney U tests. Additional subgroup analyses: obese vs non-obese (BMI ≥30), patients with vs without each clinical diagnosis (oligomenorrhoea/amenorrhoea, hirsutism, dysmenorrhoea, pelvic pain). Rotterdam phenotype distribution examined by age subgroup. | Materials and Methods, ¶9; Results (Tables 1–6) |
| 12 (c) | Explain how missing data were addressed | Missing data were handled by complete-case exclusion at the eligibility stage: patients with an incomplete hormonal laboratory panel (AMH or ≥3 hormonal parameters missing; n=156) or non-calculable BMI (n=87) were excluded prior to analysis. The final analytic cohort (n=878) had complete laboratory and anthropometric data; no imputation was used. | Materials and Methods, ¶2; Results, ¶1 |
| 12 (d) | If applicable, explain how loss to follow-up was addressed | Not applicable. All measurements were ascertained at the index diagnostic visit; there was no prospective follow-up. | N/A |
| 12 (e) | Describe any sensitivity analyses | Eight pre-specified sensitivity analyses are reported: (i) repetition of all primary analyses excluding Group 1 (≤15 yr; n=778) to address diagnostic uncertainty in early adolescents; (ii) reclassification of BMI categories in participants aged <18 years using WHO 2007 BMI-for-age z-scores, with re-computation of BMI–hormone correlations and a parallel logistic regression for hirsutism (Supplementary Table 1); (iii) repetition of all primary analyses restricted to the Rotterdam-strict subset (n=830) to address potential diagnostic misclassification in single-criterion patients; (iv) BMI-adjusted multivariable median (quantile) and log-linear regression for each primary hormonal endpoint with age subgroup as the main predictor (Supplementary Table 2); (v) restriction to an adolescent (≤19 yr) 2023 ESHRE guideline-concordant subset (concurrent oligo-/amenorrhoea AND hyperandrogenism; n=299); (vi) restriction to the strict Phenotype A subset (full Rotterdam triad; n=207) to test whether the mid-adolescent peak persists in phenotypically homogeneous patients (Supplementary Table 3); (vii) median-split temporal sensitivity analysis (early vs late study period; n=439 vs 438) to assess potential temporal heterogeneity (Supplementary Table 4); and (viii) gynaecologic-age (post-menarche) re-stratification of all primary hormonal endpoints, with multivariable median regression including chronological age, age at menarche, and BMI, to evaluate whether hormonal patterns also map to time since menarche as a more direct proxy for HPO-axis maturation (Supplementary Table 5). A parallel sensitivity logistic regression with biochemical hyperandrogenism (total testosterone >55 ng/dL) as outcome was also performed. A post-hoc Rotterdam criterion-level verification served as an additional diagnostic-validity sensitivity analysis. | Materials and Methods, ¶5, 9; Results (WHO sensitivity; Rotterdam verification; Group-1 sensitivity) |
| **Results** | | | |
| 13* (a) | Participants: Report numbers of individuals at each stage of study — eg numbers potentially eligible, examined for eligibility, confirmed eligible, included in the study, completing follow-up, and analysed | Full participant flow is reported: 11,324 source visits → 1,228 clinician-diagnosed PCOS → 350 excluded (per eligibility criteria) → 878 analytic cohort. Numbers allocated to each age subgroup (Group 1 n=100, Group 2 n=217, Group 3 n=416, Group 4 n=145) are specified. | Results, ¶1; Supplementary Figure 1 |
| 13* (b) | Give reasons for non-participation at each stage | All 10 exclusion reasons enumerated with counts: incomplete laboratory panel (n=156), non-calculable BMI (n=87), actively treated thyroid disorder (n=34), missing age/date (n=28), treated hyperprolactinaemia (n=18), 17-OHP above the non-classic CAH exclusion threshold (n=12), primary ovarian insufficiency (n=7), systemic corticosteroids (n=5), age outside 10–26 yr (n=2), duplicate record (n=1). | Materials and Methods, ¶2 |
| 13* (c) | Consider use of a flow diagram | A STROBE participant flow diagram is provided as Supplementary Figure 1, summarising the cohort derivation from source population to analytic cohort. | Supplementary Figure 1 |
| 14* (a) | Descriptive data: Give characteristics of study participants (eg demographic, clinical, social) and information on exposures and potential confounders | Table 1 provides complete demographic, anthropometric, and clinical characteristics stratified by age subgroup, including age, BMI (continuous and categorical), and clinical diagnoses (oligomenorrhoea/amenorrhoea, dysmenorrhoea, hirsutism, pelvic pain, 17-OHP ≥2.0). Table 2 reports Rotterdam criteria fulfilment and phenotype distribution. | Results; Tables 1 and 2 |
| 14* (b) | Indicate number of participants with missing data for each variable of interest | The analytic cohort (n=878) had complete laboratory and anthropometric data by design (complete-case exclusion at eligibility). This is explicitly stated in Results ¶1: "All 878 patients had complete laboratory and anthropometric panels." | Results, ¶1 |
| 14* (c) | Summarise follow-up time (eg, average and total amount) | Not applicable. Cross-sectional ascertainment at the index visit; no prospective follow-up. | N/A |
| 15* | Outcome data: Report numbers of outcome events or summary measures over time | Categorical outcomes are reported as n (%) and continuous outcomes as median [Q1–Q3] stratified by age subgroup (Tables 1, 3, 4). Key event counts: hirsutism n=114 (13.0%), oligomenorrhoea/amenorrhoea n=685 (78.0%), AMH >4.5 ng/mL n=471 across subgroups; Rotterdam ≥2 criteria met n=830 (94.5%). | Results; Tables 1, 3, 4 |
| 16 (a) | Main results: Give unadjusted estimates and, if applicable, confounder-adjusted estimates and their precision (eg, 95% confidence interval). Make clear which confounders were adjusted for and why they were included | Unadjusted between-group comparisons (Kruskal–Wallis p values with ε² effect sizes) reported for all primary and secondary parameters (Tables 3, 4). Adjusted estimates from multivariable logistic regression for hirsutism (Table 5) report β, SE, OR, 95% CI, and p for each covariate (age, total testosterone, BMI). Adjustment rationale is explained: total testosterone as the primary exposure of interest, age and BMI included as pre-specified potential confounders based on the adolescent physiology literature. | Results; Tables 3, 4, 5 |
| 16 (b) | Report category boundaries when continuous variables were categorized | BMI categories (adult WHO thresholds: <18.5, 18.5–24.9, 25.0–29.9, ≥30.0 kg/m²) and WHO 2007 z-score boundaries (thinness z<−2; overweight z>+1; obese z>+2) are explicitly defined. Age subgroup boundaries (≤15, 16–17, 18–19, ≥20) are reported. mFG ≥8 for hirsutism, 17-OHP ≥2.0 ng/mL for CAH exclusion, AMH cut-offs (>4.5 and >7.0 ng/mL), and LH/FSH >2.0 are all specified. | Materials and Methods, ¶5–7; Results (Tables 1–4) |
| 16 (c) | If relevant, consider translating estimates of relative risk into absolute risk for a meaningful time period | The odds ratio for hirsutism is reported per ng/dL of total testosterone in the Abstract (OR 1.070) and translated in the Results into an approximately two-fold increase in odds per 10 ng/dL increment, to improve clinical interpretability. Categorical prevalence estimates are provided alongside continuous predictors. | Abstract; Results (Table 5) |
| 17 | Other analyses: Report other analyses done — eg analyses of subgroups and interactions, and sensitivity analyses | The eight pre-specified sensitivity analyses listed under item 12 (e) are reported in the Results and in Supplementary Tables 1–5. Further analyses reported are: (1) within-PCOS empirical P5–P95 centile distributions by age subgroup (Table 6); (2) post-hoc Rotterdam criterion-level verification and phenotype distribution (Table 2); (3) the Phenotype D subset analysis reported in the main text; and (4) a parallel sensitivity logistic regression using biochemical hyperandrogenism as the outcome. | Results (sensitivity analyses); Tables 2, 6; Supplementary Tables 1–5 |
| **Discussion** | | | |
| 18 | Key results: Summarise key results with reference to study objectives | The opening Discussion paragraph summarises three key findings directly mapped to study objectives: (i) BMI rose across age subgroups but did not explain primary hormonal variation; (ii) the mid-adolescent hormonal peak was robust across multiple markers independent of the BMI gradient; (iii) total testosterone predicted hirsutism independently of BMI. | Discussion, ¶1 |
| 19 | Limitations: Discuss limitations of the study, taking into account sources of potential bias or imprecision. Discuss both direction and magnitude of any potential bias | A dedicated limitations paragraph enumerates: retrospective single-centre design without healthy controls; adult WHO BMI thresholds for <18-year-olds (mitigated by continuous-BMI primary analyses and WHO 2007 sensitivity analysis); BMI measurement protocol variability; absence of SHBG and FAI (direction: would likely enhance the hirsutism model per Özer et al. 2025); absence of insulin and waist circumference; baseline 17-OHP thresholds rather than ACTH stimulation testing for the 2.0–2.5 ng/mL grey zone; mFG inter-rater reliability not formally assessed; 2-year post-menarche criterion not verifiable for 8 patients; progesterone-withdrawal pharmacological stimulus in a small anovulatory subset. | Discussion (Limitations paragraph) |
| 20 | Interpretation: Give a cautious overall interpretation of results considering objectives, limitations, multiplicity of analyses, results from similar studies, and other relevant evidence | Discussion paragraphs 2–9 provide systematic interpretation: BMI-range explanation and comparison with Zabiegło et al. for adult PCOS; alignment of oligomenorrhoea/amenorrhoea prevalence with European adolescent PCOS series; mechanistic interpretation of dysmenorrhoea absence of hormonal association; reconciliation of hirsutism group-level vs patient-level findings; HPO-maturation framework for the mid-adolescent peak; comparison with 2023 ESHRE AMH caution; contextualisation of the within-PCOS centile data against published healthy-adolescent Turkish AMH reference data. Multiplicity of analyses and nominal-significance caveats are explicitly acknowledged in Methods. | Discussion, ¶2–9 |
| 21 | Generalisability: Discuss the generalisability (external validity) of the study results | Generalisability is addressed explicitly: the cohort represents the largest single-centre Turkish adolescent and young adult PCOS dataset to date; findings provide population-matched intra-PCOS benchmarks complementing (but not replacing) existing Turkish healthy-adolescent reference data. Applicability to outpatient settings lacking comprehensive androgen panels is discussed. Single-centre design limits broader generalisability, which is acknowledged. | Discussion (Limitations and Interpretation paragraphs); Conclusions |
| **Other Information** | | | |
| 22 | Funding: Give the source of funding and the role of the funders for the present study and, if applicable, for the original study on which the present article is based | The Funding statement reads: "The author(s) declare that no financial support was received for the research and/or publication of this article." | Declarations (Funding) |

**Give information separately for exposed and unexposed groups.*

*Note: An Explanation and Elaboration article discusses each checklist item and gives methodological background and published examples of transparent reporting. The STROBE checklist is best used in conjunction with this article (freely available on the websites of PLoS Medicine at http://www.plosmedicine.org/, Annals of Internal Medicine at http://www.annals.org/, and Epidemiology at http://www.epidem.com/). Information on the STROBE Initiative is available at www.strobe-statement.org.*

**Reference:** von Elm E, Altman DG, Egger M, et al. The Strengthening the Reporting of Observational Studies in Epidemiology (STROBE) statement: guidelines for reporting observational studies. J Clin Epidemiol. 2008;61(4):344-349. doi:10.1016/j.jclinepi.2007.11.008

**Supplementary File 2. RECORD Checklist**

*Reporting of studies Conducted using Observational Routinely-collected Data — Extension to STROBE*

**Manuscript: "Age-Stratified Hormonal Profiles in Adolescent and Young Adult PCOS/PMOS: Higher Mid-Adolescent AMH Largely Unexplained by BMI"**

Study design: Single-centre retrospective cohort study using Hospital Information System (HIS) data from Ankara Etlik City Hospital, Türkiye (January 2024 – February 2026).

*Reference: Benchimol EI, Smeeth L, Guttmann A, et al. The REporting of studies Conducted using Observational Routinely-collected health Data (RECORD) Statement. PLoS Med. 2015;12(10):e1001885.*

| **Item No.** | **STROBE/RECORD Recommendation** | **Compliance / Location in Manuscript** |
| --- | --- | --- |
| **Title and abstract** | | |
| **RECORD 1.1** | Indicate the study's use of routinely-collected health data and the type of data used in the title or abstract. | Abstract: "single-centre retrospective cohort... extracted from the Hospital Information System". Methods: HIS data source explicit. |
| **RECORD 1.2** | Report the type of data used (e.g., electronic health records, registry data, administrative data, claims data) in the abstract. | Methods: "Hospital Information System (HIS)" — equivalent to electronic health records. |
| **RECORD 1.3** | If linkage between databases was conducted for the study, this should be clearly stated in the title or abstract. | Not applicable. Single-source HIS data; no database linkage. |
| **Background** | | |
| **STROBE/RECORD 2** | Background, rationale, and objectives. | Introduction covers PCOS/PMOS rationale, age-stratification gap, and Turkish-population context. |
| **Methods** | | |
| **STROBE/RECORD 3** | Study design and key elements early in paper. | Methods, paragraph 1: "single-centre retrospective cohort study"; design and timeframe stated. |
| **STROBE/RECORD 4** | Setting (locations, dates). | Methods: Ankara Etlik City Hospital, January 2024 – February 2026. |
| **STROBE/RECORD 5** | Participants, eligibility, sources, methods. | Methods: ICD-10 E28.2 (PCOS) recorded at adolescent gynaecology outpatient visits; inclusion/exclusion criteria detailed. |
| **RECORD 5.1** | The methods of study population selection (such as codes or algorithms used to identify subjects) should be listed in detail. | Methods: ICD-10 E28.2 codes; consecutive adolescent gynaecology outpatient visits January 2024 – February 2026; detailed 10-category exclusion list with n for each category. |
| **RECORD 5.2** | Any validation studies of the codes or algorithms used to select the population should be referenced. | Limitations: clinician-assigned diagnosis via routine care; post-hoc Rotterdam ≥2-criterion re-abstraction confirmed in 94.5%. No external code-validation study performed; this is acknowledged in Limitations. |
| **RECORD 5.3** | If validation was conducted for this study, this should be described. | Methods: post-hoc Rotterdam criterion-level re-abstraction performed; 94.5% verified. |
| **STROBE/RECORD 6** | Variables and outcomes definitions. | Methods: hormonal panel parameters, BMI, mFG, clinical diagnoses all defined. |
| **STROBE/RECORD 7** | Data sources and measurement. | Methods: HIS extraction; all hormones on Roche Cobas e-series ECLIA (Elecsys); cycle days 2–5 sampling; standardised laboratory protocol. |
| **RECORD 7.1** | A complete list of codes and algorithms used to classify exposures, outcomes, confounders, and effect modifiers should be provided. If these cannot be reported, an explanation should be provided. | Methods: ICD-10 E28.2 for PCOS; WHO BMI categories (adult thresholds and WHO 2007 z-scores in sensitivity analysis); mFG ≥8 for hirsutism; total testosterone >55 ng/dL for biochemical hyperandrogenism; oligomenorrhoea/amenorrhoea per clinical documentation. |
| **STROBE/RECORD 8** | Bias. | Limitations paragraph: selection bias from 350 exclusions (77.4% administrative), measurement variability of BMI, mFG inter-rater reliability, assay-platform limitations all addressed. |
| **STROBE/RECORD 9** | Study size. | Methods: n=878 of 1,228 PCOS-diagnosed; all consecutive visits during 25-month period. |
| **STROBE/RECORD 10** | Quantitative variables. | Methods: continuous variables as median [IQR]; BMI both continuous and categorical analyses. |
| **STROBE/RECORD 11** | Statistical methods. | Methods Statistical Analysis section: Kruskal–Wallis, Mann–Whitney, Spearman, chi-square, logistic regression, BMI-adjusted median regression, AUROC with bootstrap CI, calibration slope/intercept; eight pre-specified sensitivity analyses; Bonferroni correction applied for primary endpoints. |
| **Results** | | |
| **STROBE/RECORD 12** | Participants, descriptive data, outcome data. | Results: Table 1 demographics; Tables 2–7 hormonal and model data; Figures 1–3 visualisations; Supplementary Tables 1–5 sensitivity analyses. |
| **RECORD 12.1** | Describe in detail the selection of the persons included in the study (i.e., study population selection) including filtering based on data quality, data availability and linkage. The selection of included persons can be described in the text and/or by means of the study flow diagram. | Methods + Supplementary Figure 1 (STROBE flow diagram): 11,324 visits → 1,228 PCOS-diagnosed → 350 excluded (10 detailed categories) → 878 analytic cohort. |
| **RECORD 12.2** | Any unforeseen events that may affect the data quality should be reported. | None reported. Single-centre, single laboratory platform (Roche Cobas e-series), uniform clinical protocol throughout 25-month study period; median-split temporal sensitivity analysis (Supplementary Table 4) confirmed temporal stability. |
| **RECORD 12.3** | Report the use of any data linkage and validation methods. | Not applicable; single-source HIS data. |
| **STROBE/RECORD 13** | Main results. | Results: peak in mid-adolescents for all four primary endpoints; BMI gradient weak; hirsutism strongest correlate is total testosterone (AUROC 0.818). |
| **STROBE/RECORD 14** | Other analyses. | Eight sensitivity analyses + post-hoc Rotterdam verification + biochemical hyperandrogenism sensitivity logistic regression. |
| **Discussion** | | |
| **STROBE/RECORD 15** | Key results. | Discussion: AMH peak robust; HPO maturation interpretation; PCOS/PMOS framework. |
| **STROBE/RECORD 16** | Limitations. | Comprehensive Limitations section: clinician-assigned diagnosis, no healthy controls, no SHBG/insulin, single-centre, immunoassay vs LC-MS/MS, no Tanner staging. |
| **RECORD 16.1** | Discuss the implications of using data that were not created or collected to answer the specific research question(s). Include discussion of misclassification bias, unmeasured confounding, missing data, and changing eligibility over time, as they pertain to the study being reported. | Limitations: HIS data not collected for research purposes; misclassification of diagnosis addressed via Rotterdam re-abstraction (94.5%); missing data addressed via exclusion of incomplete panels (77.4% of exclusions); no changes in eligibility — single-centre, single protocol; temporal sensitivity analysis confirmed stability. |
| **STROBE/RECORD 17** | Interpretation. | Discussion interprets findings as hypothesis-generating consistent with HPO maturation; explicit caution against mechanistic overclaims. |
| **STROBE/RECORD 18** | Generalisability. | Discussion: single-centre Turkish adolescent population; generalisability to other populations requires confirmation. |
| **Other information** | | |
| **STROBE/RECORD 19** | Funding. | Funding statement: no financial support was received for the research and/or publication of this article. |
| **RECORD 19.1** | Authors should provide information on how to access any supplemental information such as the study protocol, raw data, or programming code. | Data Availability Statement: de-identified aggregate data or a limited de-identified dataset may be made available from the corresponding author upon reasonable request, subject to institutional and ethics committee approval and a data-sharing agreement. Analysis code is available from the corresponding author on the same basis. |

*Note: This RECORD checklist complements the STROBE checklist (Supplementary File 1) and addresses additional reporting items specific to studies using routinely-collected health data. RECORD-specific items are marked "RECORD X.X"; items shared with STROBE are marked "STROBE/RECORD X".*
